# Chitosan-Based Gel Development: Extraction, Gelation Mechanisms, and Biomedical Applications

**DOI:** 10.3390/gels11040275

**Published:** 2025-04-06

**Authors:** Nicoleta-Mirela Blebea, Ciprian Pușcașu, Robert-Alexandru Vlad, Gabriel Hancu

**Affiliations:** 1 Department of Pharmacotherapy, Faculty of Pharmacy, “Ovidius” University of Constanța, 900470 Constanța, Romania; nicoleta.blebea@gmail.com; 2 Department of Pharmacology and Clinical Pharmacy, Faculty of Pharmacy, “Carol Davila” University of Medicine and Pharmacy, 6 Traian Vuia, 020956 Bucharest, Romania; 3 Pharmaceutical Technology and Cosmetology Department, Faculty of Pharmacy, “George Emil Palade” University of Medicine, Pharmacy, Science and Technology of Târgu Mureș, 38 Gheorghe Marinescu, 540142 Târgu Mureș, Romania; 4 Pharmaceutical and Therapeutic Chemistry Department, Faculty of Pharmacy, “George Emil Palade” University of Medicine, Pharmacy, Science and Technology of Târgu Mureș, 38 Gheorghe Marinescu, 540142 Târgu Mureș, Romania; gabriel.hancu@umfst.ro

**Keywords:** chitosan, hydrogels, gelation mechanisms, drug delivery, tissue engineering

## Abstract

Chitosan (CS), a versatile biopolymer obtained through the deacetylation of chitin, has gained significant interest in biomedical and pharmaceutical applications due to its biocompatibility, biodegradability, and unique gel-forming capabilities. This review comprehensively analyzes CS-based gel development, covering its extraction from various natural sources, gelation mechanisms, and biomedical applications. Different extraction methods, including chemical, biological, and green techniques, are discussed regarding efficiency and sustainability. The review explores the physicochemical properties of CS that influence its gelation behavior, highlighting various gelation mechanisms such as physical, ionic, and chemical cross-linking. Recent advances in gel formation, including Schiff base reactions, Diels–Alder click chemistry, and thermosensitive gelation, have expanded the applicability of CS hydrogels. Furthermore, CS-based gels have demonstrated potential in wound healing, tissue engineering, drug delivery, and antimicrobial applications, offering controlled drug release, enhanced biocompatibility, and tunable mechanical properties. The incorporation of nanomaterials, bioactive molecules, and functional cross-linkers has further improved hydrogel performance. The current review underscores the growing significance of CS-based gels as innovative biomaterials in regenerative medicine and pharmaceutical sciences.

## 1. Introduction

Marine organisms have evolved to survive in extreme environments, often developing or accumulating unique bioactive compounds as part of their adaptation strategies. These compounds, including terpenoids, sterols, alkaloids, glycosides, and peptides, exhibit distinctive chemical structures that are rarely found in terrestrial sources. Many of these substances have demonstrated significant therapeutic potential, with applications in antiviral, anticancer, anti-inflammatory, antidiabetic, neuroprotective, and immunomodulatory treatments. Consequently, the marine ecosystem has become a valuable reservoir for drug discovery [1,2,3].

Polysaccharides, also known as glycans, are complex carbohydrates composed of multiple monosaccharides. Their structural diversity arises from variations in sugar composition, glycosidic linkage patterns, branching, chain length, and molecular weight [4]. Based on their composition, polysaccharides are classified into homopolysaccharides, which consist of a single type of sugar monomer (e.g., glucose, fructose, galactose, or chitin), and heteropolysaccharides, which contain multiple sugar types. Homopolysaccharides are widely distributed across algae, animals, microorganisms, and plants, where they play essential roles in cell signaling and immune regulation. In contrast, heteropolysaccharides, such as glycoproteins and mucopolysaccharides, are predominantly found in animal tissues, bacterial cell walls, and extracellular matrices [5].

The exploration of natural polysaccharides from marine sources has significantly expanded in recent years, with seaweed recognized as the most abundant producer. Bioactive polysaccharides such as alginates, carrageenan, and fucoidans have been extensively studied for their functional properties and potential health benefits [6,7,8]. However, polysaccharides derived from marine animals hold great promise for biomedical and industrial applications. These polysaccharides can be classified into two primary categories:

- Gel-forming polysaccharides—compounds characterized by high osmotic pressure, viscosity, and water-absorbing capabilities, making them valuable in pharmaceuticals, drug delivery systems, plasma substitutes, and biomedical nanomaterials [9,10].
- Biologically active polysaccharides—known for their diverse health benefits, these molecules exhibit antioxidant, antiobesity, antiparasitic, and metabolic syndrome-modulating properties [11].

The major marine animal polysaccharides are divided into:

- Glycosaminoglycans and mucin-type O-glycans, which are essential for maintaining intestinal health [12].
- Sulfated polysaccharides, found in sponges, sea cucumbers, and starfish, distinguished by their high sulfate content.
- Chitin, extracted primarily from crustaceans like shrimp and crabs, widely utilized in biomaterial research.
- Acid mucopolysaccharides, present in several marine organisms and exhibiting notable bioactive properties [11].

The extreme conditions of the marine environment, including high salinity, elevated pressure, and low light availability, have led to the evolution of polysaccharides with enhanced charge density, higher sulfate content, and unique molecular weight distributions [13]. These features contribute to their broad applications in the food, cosmetic, and pharmaceutical industries, where they are used as functional ingredients and biomaterials [14]. Chitosan (CS), a deacetylated derivative of chitin, stands out due to its biocompatibility, biodegradability, adhesive properties, and unique gel-forming abilities [15].

Building on the remarkable properties of marine-derived polysaccharides, this review explores the development of CS-based gels, highlighting their potential as versatile biomaterials. With its biocompatibility, controlled gelation behavior, and broad applicability, CS has gained increasing attention in pharmaceutical and biomedical research. By examining advancements in extraction techniques, gel formation mechanisms, and medical applications, this work aims to underscore the growing significance of CS hydrogels in areas such as drug delivery, wound healing, and tissue engineering.

This review explores the development of CS-based gels, with a particular focus on their extraction methods, physicochemical characteristics, gelation processes, and biomedical uses. By analyzing recent progress and current trends in CS gel formulations, the review emphasizes the growing importance of marine-derived polysaccharides in creating innovative biomaterials for pharmaceutical and medical applications.

While numerous reviews have addressed CS-based hydrogels, the present work offers a distinct and comprehensive perspective by integrating the entire developmental pipeline, from raw material sourcing and extraction methods to cross-linking mechanisms and advanced biomedical applications. Furthermore, we place particular emphasis on environmentally friendly extraction techniques and green chemistry approaches. By combining structural, chemical, and functional insights, this review aims to serve as both a scientific synthesis and a practical guide for future research and clinical translation of CS-based hydrogels.

## 2. Chemical Structure

CS is obtained through the deacetylation of chitin, a naturally abundant polysaccharide found in the exoskeletons of crustaceans, insects, and fungal cell walls. Structurally, chitin is composed of β-(1→4)-linked N-acetylglucosamine units, and its partial deacetylation results in CS, which contains both glucosamine and N-acetylglucosamine residues. This structural modification significantly improves the solubility of CS in acidic solutions, as the removal of acetyl groups increases the proportion of amine (-NH_2_) functional groups, enhancing its reactivity and versatility in various applications [16].

CS shares a close structural relationship with cellulose, which consists of β-(1→4)-linked *D*-glucose units [17]. However, in chitin and CS, the hydroxyl (-OH) group at the C-2 position in cellulose is replaced by an acetamide group, a characteristic feature that distinguishes them. Chemically, CS is a β-(1→4)-linked 2-amino-2-deoxy-β-*D*-glucopyranose, though it retains some acetamide groups due to incomplete deacetylation [18,19]. One of the key differences between cellulose and CS is the nitrogen content. CS contains approximately 5%–8% nitrogen, present as acetylated -NH_2_ groups in chitin and as primary -NH_2_ groups in CS, which confer unique reactivity and functionalization potential [20].

**Figure 1:**
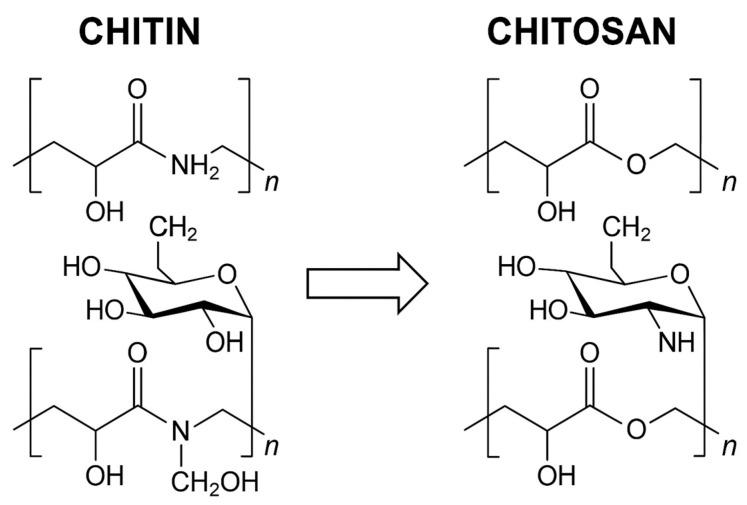
Structural comparison between chitin and chitosan. Chitin is composed of β-(1→4)-linked N-acetyl-*D*-glucosamine units, while chitosan results from the partial deacetylation of chitin, replacing the acetyl group (-COCH_3_) at the C2 position with a primary amino group (-NH_2_) (created with Biovia Draw, version 2025, Dassault Systèmes, Vélizy-Villacoublay, France by Hancu G.).

Structurally, chitin is composed of β-(1→4)-linked N-acetylglucosamine units, and its partial deacetylation yields chitosan, which contains both glucosamine and N-acetylglucosamine residues. This deacetylation process replaces the acetyl groups at the C_2_ position with primary -NH_2_ groups, enhancing the solubility and chemical reactivity of the polymer. A visual comparison of chitin and chitosan structures is provided in Figure 1, illustrating the key chemical differences that underpin their distinct physicochemical and biological properties.

The primary and secondary -OH groups in CS, alongside its -NH_2_ groups, make it significantly more chemically active than its precursor, chitin. These functional groups enable a wide range of chemical modifications, allowing researchers to modify its mechanical strength, solubility, and biological properties for specific applications [21].

## 3. Physicochemical Properties

CS is a linear cationic polysaccharide that typically appears as a white, flake-like, or powdery solid with a slight pearlescent sheen. It is widely recognized for its biodegradability, biocompatibility, and non-toxic nature, making it an attractive material for biomedical and pharmaceutical applications [21,22]. Due to its rigid *D*-glucosamine structure, CS exhibits high hydrophilicity and crystallinity, which enables the formation of intermolecular hydrogen bonds and contributes to its high viscosity. The presence of reactive functional groups, particularly -NH_2_ and -OH groups, allows for cross-linking and various chemical modifications, expanding its range of applications [23].

It is insoluble in most organic solvents, but can be dissolved in diluted acidic solutions, such as hydrochloric acid, formic acid, and acetic acid [21,22]. In acidic conditions, the -NH_2_ groups interact with hydrogen protons, forming a positively charged polyelectrolyte. This interaction disrupts the hydrogen bonding network between CS molecules, thereby increasing its solubility in water. The solubility of CS is primarily influenced by molecular weight and the degree of deacetylation (DD). More deacetylation increases the protonation of -NH_2_ groups, enhancing solubility, whereas a higher molecular weight promotes intramolecular and intermolecular hydrogen bonding, leading to chain entanglement and reduced solubility [24].

Although an ideal CS structure would be fully deacetylated, achieving complete deacetylation is challenging. In practice, commercially available CS exhibits varying DD and molecular weights, which depend on the source material and production methods. Typically, CS with a DD above 55% is soluble in a 1% nitric acid solution [25].

CS is a weak base with a pKa of approximately 6.3. Its deprotonated -NH_2_ groups act as strong nucleophiles, making it highly reactive in various chemical modifications. CS forms salts with both organic and inorganic acids, demonstrating excellent chelating and complexing abilities. Additionally, it exhibits ionic conductivity, which further enhances its applications in drug delivery and treatment of water [23]. As a cationic biopolymer with high charge density, CS interacts strongly with negatively charged molecules, making it an effective flocculating agent. Moreover, its film-forming ability and adhesive properties contribute to its usefulness in biomolecule isolation, immobilization, and controlled drug release [23].

CS exhibits polymorphism, with three primary crystalline forms identified by Jang et al. [26]:

- α-form CS: most common form, characterized by a highly ordered structure with two antiparallel polysaccharide chains, leading to strong intermolecular hydrogen bonding and high crystallinity [27].
- β-form CS: composed of two parallel polysaccharide chains, exhibiting weaker hydrogen bonding and reduced crystallinity compared to the α form [28].
- γ-form CS: a more complex structure, consisting of three parallel chains, with two aligned in one direction and the third oriented oppositely [29].

The crystallinity of CS is influenced by its DD and molecular weight, both of which dictate its mechanical strength, solubility, and interaction with other compounds [30].

The viscosity of CS solutions depends on multiple factors, including deacetylation level, molecular weight, and concentration. As the DD and molecular weight increase, so does viscosity due to higher chain entanglement and stronger intermolecular interactions. However, temperature and acidic storage conditions can significantly affect viscosity. In acidic solutions, CS undergoes degradation, leading to a reduction in molecular weight and viscosity over time. Notably, in 0.1 M acetic acid, the viscosity of CS decreased from 7.4 to 2.2 dL/g within 48 h due to the acidic hydrolysis of glycosidic bonds [31].

**Figure 2:**
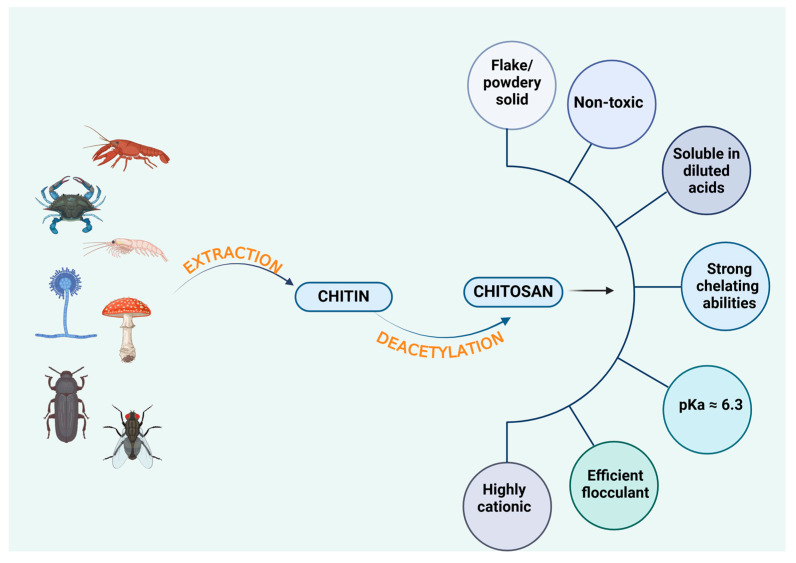
CS production from chitin: sources, extraction, and properties. Created with BioRender (web application, accessed February 2025; BioRender Inc., Toronto, ON, Canada; www.biorender.com). Figure prepared by Pușcașu C.

The key physicochemical properties of CS are summarized in Figure 2, which illustrates its solubility profile, crystallinity, chemical reactivity, and overall structural characteristics.

## 4. Sources of Chitosan

CS is primarily obtained from crustacean shells, such as shrimp, crab, and lobster, which remain the dominant commercial source due to their abundance and high chitin content [32]. However, alternative sources are gaining interest, particularly from the perspectives of sustainability, biocompatibility, and unique physicochemical properties. These alternative sources include fish scales, fungi, and insects, each offering distinct advantages in terms of resource availability, extraction efficiency, and environmental impact (Figure 2).

### 4.1. Crustacean-Derived Chitosan

Annually, the global seafood industry generates approximately 18 to 30 million tons of fish waste, posing significant environmental risks due to its high biological oxygen demand, chemical oxygen demand, total suspended solids, pathogens, and organic matter accumulation [33]. However, despite these environmental concerns, fish waste represents a valuable resource, particularly in the production of CS.

Currently, shrimp, crab, and krill serve as the primary commercial sources of CS, as their exoskeletons are available as by-products of seafood processing industries [32]. However, chitin is embedded within a complex matrix of proteins and minerals, necessitating demineralization and deproteinization as crucial steps in the extraction process [34]. The final deacetylation step converts chitin into CS, with shrimp shells being the most preferred source due to their higher extraction efficiency and physicochemical properties that closely resemble commercially available CS [35].

While shellfish-derived CS offers significant advantages, such as high chitin content, ease of extraction, and commercial availability, it also presents certain challenges. One of the major concerns is allergenicity, as CS extracted from shellfish may trigger allergic reactions in sensitive individuals, limiting its applications in pharmaceutical and biomedical fields. Additionally, the extraction process often involves harsh chemical treatments, particularly strong acids and bases used for demineralization and deproteinization, which can lead to environmental concerns related to waste disposal and pollution [36,37,38].

### 4.2. Fish-Derived Chitosan

Fish scales, often discarded as waste, account for approximately 1% of a fish’s total weight and are a significant contributor to river pollution due to their slow biodegradability and accumulation in aquatic ecosystems. Studies have shown that fish scales can yield up to 37.4% CS after dehydration, making them a promising alternative source [39].

Beyond CS extraction, fish scales have demonstrated remarkable functional properties in various applications. Liaw et al. [40] found that hydroxyapatite CS composites derived from fish scales exhibit high efficiency in removing heavy metal ions from wastewater. Their adaptive porous structure enhances adsorption capacity, allowing them to function effectively in both static and dynamic environments. This versatility highlights their potential for environmental and industrial applications, particularly in water purification, bioremediation, and development of sustainable materials.

### 4.3. Fungus-Derived Chitosan

Fungal cell walls, much like crustacean shells, contain chitin, making fungi the second-largest natural source of chitin after crustaceans. Chitin typically constitutes 1%–15% of the fungal biomass, depending on the species and growth conditions [41]. Unlike crustacean-derived chitin, fungal chitin does not require harsh acid treatments for demineralization and purification, significantly simplifying the extraction process and reducing environmental impact [42].

Beyond its structural role, CS serves as a natural defense modulator in plants, enhancing disease resistance and growth promotion. Additionally, studies have shown that fungus- and crab-derived CS exhibits stronger free radical scavenging activity compared to CS extracted from insects and shrimp, highlighting their potential antioxidant properties [39,43].

Certain fungal species, particularly those belonging to the *Aspergillus* and *Mucor* genera, are well-known for their ability to produce chitin-rich cell walls, making them ideal candidates for fungus-derived CS production. Despite these advantages, fungus-derived CS has limitations. Its lower chitin content results in a lower CS yield per biomass, making the extraction process less efficient than that of crustacean-derived CS. Additionally, fungal cultivation and CS extraction require specialized fermentation techniques, which can be more complex, resource-intensive, and time-consuming compared to conventional crustacean extraction methods [44,45,46].

### 4.4. Insect-Derived Chitosan

Insects, including beetles, ants, butterflies, and various other arthropods, contain chitin in their exoskeletons, making them a promising alternative source of CS. Unlike traditional sources such as crustaceans, insects reproduce rapidly, require minimal space and feed, and can be cultivated on organic waste, making them a viable option for large-scale CS production [41,47,48,49,50].

One of the most significant advantages of insect-derived CS is the diversity of chitin compositions across different species, which allows for variations in polymer characteristics such as molecular weight, DD, and physicochemical properties. This diversity broadens the potential applications of insect-derived CS in biomedicine, food preservation, pharmaceuticals, and environmental remediation. However, large-scale production remains challenging due to limited biomass availability and potential social and cultural resistance to using insects as raw materials in industrial and biomedical applications. Public perception, regulatory frameworks, and consumer acceptance are still major obtacles to the widespread adoption of insect-derived CS [41,47,48,49,50].

The chitin yield from insects varies significantly among species, influencing the overall efficiency of CS production. Mohan et al. [51] reported that *H. parallela* and *T. molitor* yield 15% and 17.32% chitin, respectively, with *T. molitor* (mealworm beetle) being particularly suited for low-cost artificial breeding, making it an economically viable candidate for industrial CS production. Additionally, several other insect species have demonstrated noteworthy chitin and CS yields: *B. mori* (silkworm), *E. kuehniella* (Mediterranean flour moth), *D. punctatus* (pine caterpillar moth), *A. pandora* (butterfly species), and *C. bilineata* (hawkmoth species). Chitin yields from these species range from 2.59% to as high as 96.2% dry weight, depending on the species, developmental stage, and extraction methods. The ease of insect cultivation, short life cycles, and high reproductive rates further contribute to their growing potential as a sustainable and scalable source of CS.

Table 1 summarizes various biological sources of chitin along with their chitin yield percentages, highlighting their potential for CS production.

## 5. Extraction Methods

**Figure 3:**
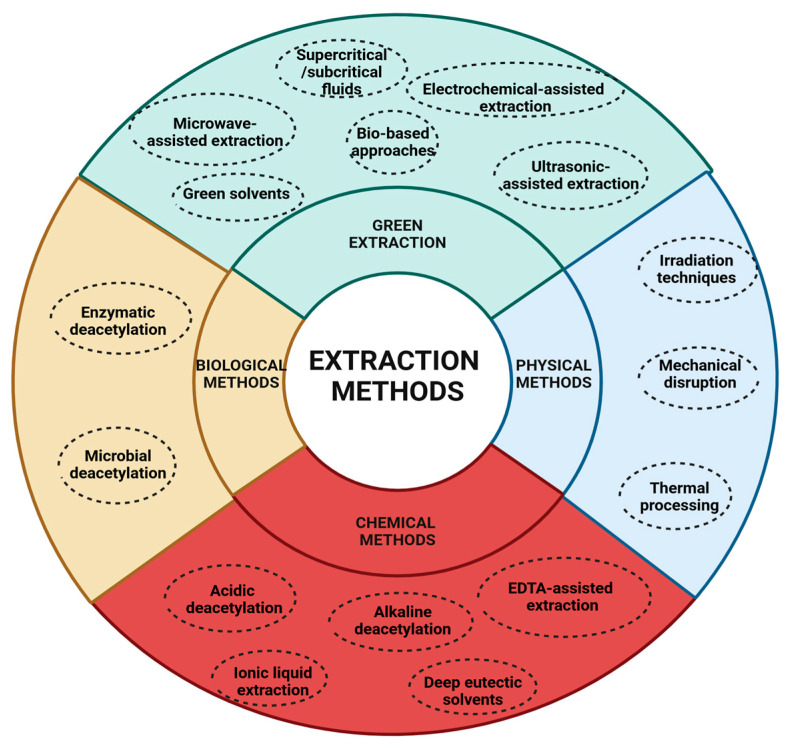
Extraction methods of CS (EDTA—ethylenediaminetetraacetic acid); Created with BioRender (web application, accessed February 2025; BioRender Inc., Toronto, ON, Canada; www.biorender.com). Figure prepared by Pușcașu C.).

CS can be extracted from chitin using physical, chemical, biological, or green methodologies, as illustrated in Figure 3. These techniques may be employed independently or in combination to enhance efficiency and maximize yield.

### 5.1. Physical Methods

Physical methods alter chitin’s structure to enhance deacetylation and can be applied alone or in combination with chemical and biological approaches to improve efficiency.

1. Irradiation Techniques
  - Microwave-assisted deacetylation [67].
  - UV irradiation: employs high-energy ultraviolet radiation to break down chitin, making it more susceptible to deacetylation [68].
2. Mechanical Disruption
  - Grinding/milling: reduces chitin into finer particles, increasing its surface area and facilitating deacetylation reactions [69].
  - Sonication: uses ultrasonic waves to generate microcavitation within the chitin structure, improving the penetration of deacetylating agents [70].
3. Thermal processing
  - The application of heat accelerates chitin deacetylation by increasing its reactivity. However, excessive temperatures may lead to CS degradation, potentially compromising its quality [71].

Physical methods enhance chitin reactivity, thereby improving deacetylation efficiency. However, they require significant energy input and may alter chitin’s structure. Consequently, they are typically used in combination with chemical or biological methods rather than as standalone approaches [72].

### 5.2. Chemical Methods

Conventional chemical methods for CS extraction rely on strong alkali solutions to accelerate deacetylation, removing acetyl groups from chitin to produce CS. The two primary approaches are as follows.

- Acidic deacetylation. Chitin is treated with acids such as HCl or H_2_SO_4_ to disrupt its crystalline structure, increasing reactivity. However, this method is less effective in achieving complete deacetylation and may lead to low-molecular-weight CS due to acid hydrolysis of the aminopolysaccharide backbone [73,74].
- Alkaline deacetylation. Chitin is treated with strong bases like NaOH or KOH at high temperatures for extended periods. This process cleaves acetyl–amino linkages, releasing acetate ions and converting chitin into CS [75].

To improve product quality and reduce environmental impact, alternative chemical extraction methods have been explored, as follows.

- EDTA-assisted extraction. Ethylenediaminetetraacetic acid (EDTA) helps remove inorganic salts by forming complexes with calcium ions [76]. Zhang et al. [39] demonstrated that EDTA-assisted extraction improved chitin’s crystallinity and degree of acetylation (DA) compared to conventional acid treatments [77]. Despite better working conditions and improved product quality, EDTA extraction still requires alkali for protein removal and shares limitations such as high cost, chemical use, and challenges in waste treatment [78].
- Ionic liquid extraction. Ionic liquids, organic salts in a liquid state below 100 °C, offer a tunable, recyclable, and low-volatility medium for chitin dissolution and deacetylation [79]. Ionic liquids can disrupt hydrogen bonds in chitin, enabling extraction without harsh chemicals [34]. Studies using ammonium-based ionic liquids showed high selectivity and extraction efficiency from shrimp shells. Ionic liquids can assist in demineralization, deproteinization, and even deacetylation steps [80]. However, current research on ionic liquids is limited regarding their deacetylation performance, toxicity, and safety, which restricts their industrial application [78].
- Deep eutectic solvents. Deep eutectic solvents are mixtures of hydrogen bond donors and acceptors that create a eutectic system with a melting point lower than either component [81]. Deep eutectic solvents like choline chloride–lactic acid have been used to extract high-purity chitin (up to 99.3%) in shorter times while also showing low phytotoxicity [82,83]. Deep eutectic solvents are biodegradable, non-toxic, thermally stable, and easy to prepare, making them promising green alternatives to traditional solvents. Compared to ionic liquids, deep eutectic solvents are considered safer and more environmentally friendly. However, large-scale application is still limited due to a lack of industrial studies and long-term safety data [78].

Chemical methods, particularly alkaline deacetylation, are widely used due to their efficiency and high deacetylation yields. However, they require significant chemical and energy input, generate waste, may degrade CS, and lack selectivity, resulting in a random acetyl group distribution [84].

### 5.3. Biological Methods

Biological methods employ enzymes and microorganisms to naturally deacetylate chitin, offering a sustainable alternative to chemical processes, as follows.

- Enzymatic deacetylation. Chitinase and related enzymes, produced by bacteria, fungi, and plants, break glycosidic bonds in chitin, while chitosanase further deacetylates CS, modifying its properties [85]. This method is environmentally friendly and highly selective, but tends to be slower and more expensive due to enzyme costs and the need for controlled conditions [86].
- Microbial deacetylation. Certain bacteria (*S. marcescens*, *P. aeruginosa*) and fungi (*A. niger*, *M. rouxii*) naturally produce enzymes that deacetylate chitin. This process operates under near-ambient conditions, reducing energy consumption. However, it is slower, requires specific growth conditions, and adds complexity to the extraction process [87].

Biological methods offer an eco-friendly alternative to chemical deacetylation, requiring less energy and producing minimal waste. They enable selective deacetylation for tailored CS properties, but remain limited by slower reaction rates and higher costs due to enzyme expenses or specialized microbial growth requirements [88].

### 5.4. Green Extraction Methods

Green extraction methods minimize environmental impact by reducing harsh chemicals and energy use, as follows.

- Supercritical/subcritical fluids. These utilize fluids like supercritical CO_2_ or water for deacetylation, enhancing purity and process efficiency [89].
- Green solvents. Ionic liquids dissolve and deacetylate chitin under mild conditions, reducing chemical waste. However, challenges in solvent recovery and reuse remain a limitation [90].
- Bio-based approaches. Enzymes or microorganisms deacetylate chitin with minimal waste, aligning with sustainable biorefinery principles [91].
- Microwave-assisted extraction. This accelerates deacetylation while significantly reducing energy consumption [92]. It achieves rapid, uniform heating through dipolar polarization and ionic conduction. Microwave-assisted extraction can reach ~82% deacetylation in just 24 min compared to 6–7 h with conventional methods while maintaining similar product quality. It also improves deproteinization and demineralization and allows control over molecular weight. Optimizing parameters like power, time, solvent concentration, and solid-to-liquid ratio is essential [90].
- Ultrasound-assisted extraction. This utilizes ultrasound-induced cavitation to enhance the solubility of proteins and depolymerize chitin macromolecules, improving extraction efficiency. High-intensity ultrasound (750 W, 20 kHz) reduces extraction time and avoids high temperatures [93]. Ultrasound-assisted extraction is effective as a pretreatment to reduce chitin crystallinity, improving enzymatic hydrolysis. Combined with steam explosion, it significantly lowers the DA without extensive depolymerization [94]. Applying ultrasound in cycles (7.5 min on, 5 min off) further improves chitosan yield, achieving a DA as low as 22.1% [95].
- Electrochemically assisted extraction. This involves electrolysis using inert electrodes and low-molecular-weight salt solutions (NaCl, Na_2_SO_4_) to facilitate decellularization, demineralization, and purification steps. By controlling pH through electrode reactions, proteins, pigments, and minerals are removed without harsh chemicals. The method was effectively used to isolate pure 3D sponge chitin, confirmed by N-acetylglucosamine content and chitinase digestion [96].

Green extraction methods help reduce the environmental footprint of CS production by limiting the use of hazardous chemicals and lowering energy demands. While they yield high-purity CS, their implementation often requires specialized equipment or materials (e.g., green solvents, supercritical fluids) and still faces challenges in efficiency and scalability [90]. Table 2 provides a summary of the key advantages and disadvantages associated with different chitin extraction methods for CS production.

## 6. Main Applications of Chitosan

Owing to their biocompatibility, biodegradability, safety, and diverse biological activities, CS and its derivatives have attracted considerable attention, particularly in biomedical, food, biotechnology, and pharmaceutical applications [97,98].

### 6.1. Biomedical Applications of Chitosan

Several biomedical applications have been considered in the literature and are described in the following sections of this review.

#### 6.1.1. Hemostatic and Cardiovascular Effects

CS interacts with blood cells via its -NH_2_ groups, inducing clot formation through thrombogenic or hemolytic responses. This process begins with plasma adsorption onto CS, followed by platelet adhesion and activation, ultimately leading to thrombus formation. Sulfation of CS’s -OH or -NH_2_ groups has been shown to enhance its anticoagulant, antioxidant, antimicrobial, and hemagglutination-inhibiting properties.

Lih et al. developed a CS–polyethylene glycol–tyramine bioadhesive hydrogel that formed rapidly in situ using horseradish peroxidase and hydrogen peroxide. It effectively stopped liver bleeding in rats, combining CS’s hemostatic properties with strong tissue adhesion. The hydrogel formed within 5 s upon contact with skin. Histological analysis showed superior wound healing compared to fibrin glue, sutures, and cyanoacrylate, highlighting its potential for rapid and effective wound closure [99].

Singh et al. developed a hydrogel dressing using quaternized and phosphorylated CS, with tannic acid serving as both a hemostatic agent and cross-linker. The addition of poly-ε-lysine enhanced the system’s elasticity and adhesion, achieving an adhesion strength of 0.00915 MPa, twice that of gelatin and three times that of commercial Axiostat. Hematological and serum tests showed no signs of inflammation or toxicity in rats. In vivo studies demonstrated superior hemostatic performance, with faster clotting compared to Axiostat (approximately 225 s), confirming its potential as an effective wound dressing [100].

Beyond its medical applications, CS also exhibits hypocholesterolemic and hypolipidemic effects, contributing to reduced cardiovascular risk [101]. CS oligomers with low molecular weight were found to be more effective in enhancing lipoprotein lipase activity in the liver and plasma of mice. In contrast, oligomers with a higher DD significantly increased cholesterol levels and fecal fat excretion, indicating differing metabolic effects based on their structural properties [102].

#### 6.1.2. Antimicrobial Properties

CS, its derivatives, and chitooligosaccharides (COSs) exhibit antimicrobial activity in vitro against a range of microorganisms, including *A. hydrophila*, *S. aureus*, *E. coli*, *P. aeruginosa*, *L. monocytogenes*, *S. typhimurium*, and *V. cholerae* [103,104,105]. CS has shown strong inhibitory effects against *C. albicans*, *C. krusei*, and *C. glabrata*, whereas other derivatives, such as carboxymethyl CS (CMCS) and COS, exhibit weaker or no antimicrobial activity [53,106].

CS-based wound dressings have proven effective in combating *P. aeruginosa*, *B. cereus*, and *L. monocytogenes*, playing a crucial role in wound healing, hemostasis, and infection control [22,107,108]. Ling et al. developed an eco-friendly antibacterial paper using a nanocomposite of silver nanoparticles loaded onto quaternized CMCS and organo-modified montmorillonite. The resulting paper exhibited strong antimicrobial activity, with surface coating proving more effective than internal additive methods in enhancing its antibacterial performance [109].

To develop antibacterial implantable biomaterials for clinical use, genipin-cross-linked, gentamycin sulfate-loaded CMCS hydrogels were created. These hydrogels effectively inhibited *S. aureus* growth and biofilm formation while also enhancing adhesion, proliferation, and differentiation of osteoblastic MC3T3-E1 cells. The hydrogel’s positive charge disrupted bacterial membranes and supported cell activity. Genipin concentration influenced both degradation rate and cell response, while gentamycin loading further boosted antibacterial and osteogenic effects [110].

A zinc oxide–CMCS bionanocomposite was developed as an antibacterial and anti-ultraviolet coating for cotton fabric. The treated fabric showed strong antibacterial activity against both Gram-positive and Gram-negative bacteria, along with effective UV protection. Increasing the curing temperature further enhanced the UV-blocking ability of the coated fabric [111].

Despite extensive research, the precise antimicrobial mechanism of CS, COS, and their derivatives remains a topic of debate due to differences in polymer composition, purity, microbial strains, and experimental methodologies. One proposed mechanism suggests that CS reduces cell membrane permeability by allowing its -NH_2_ groups to interact with microbial -COOH groups, thereby restricting nutrient transport. The DA significantly influences this activity. Another hypothesis posits that CS can penetrate bacterial cells and interfere with RNA transcription by adsorbing bacterial DNA, ultimately disrupting cellular processes [112].

#### 6.1.3. Antioxidant Activity

CS and its derivatives possess strong antioxidant properties, playing a crucial role in tissue repair, scavenging excess free radicals, enhancing antioxidant enzyme activity, and inhibiting lipid peroxidation. These effects contribute to delaying the aging process. Studies suggest that the antioxidant activity of CS is closely associated with its molecular weight and DD, with lower molecular weight and higher DD generally enhancing its antioxidant potential [113,114]. The antioxidant capacity of CS is believed to stem from its functional groups. Its -OH groups can interact with hydroxyl radicals (OH•) through hydrogen atom donation, while residual free -NH_2_ groups can form stable macromolecular free radicals upon reacting with OH•. Additionally, in aqueous solutions, -NH_2_ groups can undergo protonation to form NH_3_^+^, which subsequently reacts with OH• through an addition reaction [115,116].

Ferulic acid, caffeic acid, and gallic acid were grafted onto N,O-CMCS, and the resulting conjugates were evaluated for antioxidant activity. The antioxidant effectiveness decreased in the following order: CS > ferulic acid-grafted N,O-CMCS > caffeic acid-grafted N,O-CMCS > gallic acid-grafted N,O-CMCS [117]. CMCS modified with silk peptide showed notable antioxidant activity, with maximum scavenging rates of 24.86% for 2,2-diphenyl-1-picrylhydrazyl, 91% for hydroxyl radicals, and 36.8% for hydrogen peroxide. Cytotoxicity tests using NIH-3T3 mouse fibroblasts confirmed that the CMCS modified with silk peptide copolymers was non-toxic, indicating its potential for safe biomedical applications [118].

#### 6.1.4. Antitumor Activity

Recent studies indicate that CS and its derivatives exhibit antitumor activity, primarily by inhibiting tumor cell proliferation and inducing apoptosis. In vitro research on B16 melanoma cells has demonstrated their effectiveness in triggering programmed cell death. Additionally, CS has been shown to enhance immune system activation, facilitating tumor elimination in a B16F10 melanoma tumor-bearing mouse model [119]. Seleno-short-chain CS has also been found to induce apoptosis in human gastric cancer BGC-823 cells by activating the mitochondrial signaling pathway. This mechanism involves the disruption of mitochondrial membrane potential, excessive accumulation of reactive oxygen species, an increased Bax/Bcl-2 ratio (Bcl-2-associated X protein/B-cell lymphoma 2 protein), and the activation of caspase 3, caspase 9, and cytochrome C [120].

Another study demonstrated that CS nanoparticles (CNPs) bind to the CD44 receptor, improving targeted delivery and enhancing drug accumulation in tumor cells. Notably, hyaluronic acid (HA)-coated CNPs localize within mitochondria, where they generate high levels of reactive oxygen species and activate the mitochondrial apoptotic pathway, thereby strengthening their synergistic antitumor effect. This study was conducted in vitro using A549 cells (a human lung carcinoma cell line with high CD44 expression) and HepG2 cells (a liver cancer cell line with low CD44 expression) [121]. In vivo experiments further revealed that polymer–drug conjugates, such as CMCS and norbornene, reduce systemic toxicity while enhancing antitumor efficacy. In BALB/c nude mice bearing SGC-7901 gastric tumors, these conjugates achieved a tumor suppression rate of 59.57%. Mechanistically, they upregulated pro-apoptotic tumor necrosis factor alpha (TNF-α) and Bax while downregulating vascular endothelial growth factor (VEGF), Bcl-2, matrix metalloproteinase 2, and matrix metalloproteinase 9. These effects collectively induced apoptosis and inhibited tumor metastasis [122].

The antitumor metastasis effect of CMCS was evaluated using mouse hepatoma 22 cells and human liver cancer cells (BEL-7402). CMCS significantly inhibited tumor cell migration in vitro and reduced matrix metalloproteinase 9 expression in a dose-dependent manner. In vivo, it strongly suppressed lung metastasis in mice and improved lung damage caused by tumor spread. These effects were partly linked to decreased levels of E-selectin and vascular endothelial growth factor in CMCS-treated mice, highlighting its potential as an anti-metastatic agent [123]. Further, a CMCS–quercetin conjugate with amphiphilic properties was synthesized to enhance the oral delivery of the anticancer drug paclitaxel. The paclitaxel-loaded micelles showed sustained release in various pH conditions and significantly increased intestinal absorption and permeability compared to verapamil. In tumor models, the micelles improved oral bioavailability and demonstrated strong antitumor activity with a better safety profile than standard paclitaxel formulations [124].

The oral bioavailability of tamoxifen was enhanced using α-tocopherol succinate-grafted CMCS micelles. These tamoxifen-loaded polymeric micelles showed improved permeability, reduced liver toxicity, and strong anticancer activity. The micelles efficiently accumulated in the cytosol of MCF-7 breast cancer cells and significantly outperformed pure tamoxifen in suppressing tumors in a mammary carcinoma rat model, highlighting their potential for safer and more effective breast cancer therapy [125].

CS-based nanocarriers hold strong potential for clinical drug delivery, particularly in cancer treatment, due to their natural origin, biodegradability, biocompatibility, and modifiability. However, challenges such as hemocompatibility, off-target drug release, and possible toxicity from chemical modifications must be addressed. Although CS is FDA-approved for applications like wound healing and oral delivery, each formulation requires thorough safety evaluation [126].

Practical limitations including scalability, production consistency, and regulatory complexity also hinder clinical translation. Nevertheless, CS’s sustainable sourcing from crustacean shells supports circular economy goals [127].

Future research should prioritize designing stimuli-responsive nanoparticles that adapt to tumor microenvironments using triggers like redox conditions, temperature, magnetic fields, or ultrasound. Optimizing size, surface properties, circulation time, and deformability is critical for targeted delivery and therapeutic performance [128].

In addition to these considerations, potential immune responses and degradation behavior also present challenges for CS-based nanocarriers in cancer therapy. Depending on molecular weight and DD, CS may induce immune reactions, which can affect therapeutic outcomes [129]. Moreover, CS’s enzymatic degradation in vivo can be unpredictable and may compromise the stability and controlled release of the drug [130,131].

While CS shows promise in cancer therapy, some challenges remain. These include possible immune reactions, variability in degradation rates, and difficulties with targeted delivery. Inconsistent properties between batches can also affect reproducibility. Further research is needed to improve stability, biocompatibility, and tumor specificity before clinical use.

#### 6.1.5. Anti-Inflammatory Properties

CS’s anti-inflammatory effects are closely associated with its ability to reduce free radicals, particularly in its lower-molecular-weight form COS. In a lipopolysaccharide-induced RAW 264.7 macrophage model, medium- and low-molecular-weight CS effectively inhibited nitric oxide (NO) production, whereas COS exhibited the opposite effect. The underlying mechanisms differ, with complement receptor 3 and Toll-like receptor 4 playing critical roles in regulating the nuclear factor kappa-light-chain enhancer of activated B cells and inducible nitric oxide synthase pathways [132].

Moreover, CS can reduce inflammatory responses by binding to antigens and lipopolysaccharides, playing a key role in modulating immune-related inflammation. Studies show that the lipopolysaccharide–CS complex is 10–20 times less toxic than lipopolysaccharide alone, mainly due to electrostatic interactions between the polycationic CS and the negatively charged phosphate groups in lipopolysaccharide [133].

Additionally, CS can influence macrophage polarization. Research has shown that chitosan scaffolds with a low DA (5%) promote the expression of anti-inflammatory cytokines and encourage M2 macrophage polarization, which is associated with tissue repair. In contrast, CS with a higher DA (15%) tends to induce a pro-inflammatory response, favoring M1 macrophage polarization [134].

#### 6.1.6. Tissue Engineering

CS is extensively used as a polymer scaffold in tissue engineering due to its high porosity, biodegradability, structural integrity, and biocompatibility. These properties promote cell adhesion and support various cellular functions [135]. Tissue-engineered materials incorporating CS have demonstrated promising applications in cartilage regeneration, nerve repair [136], bone healing [137], and tracheal tissue engineering [138].

Prakash et al. developed CS–polyvinyl alcohol films incorporating a graphene oxide, hydroxyapatite, and gold hybrid for potential orthopedic use. The composite was synthesized via hydrothermal and gel casting methods. The films showed high biocompatibility with minimal red blood cell lysis (3.74%) and promoted osteoblast activity, indicated by increased alkaline phosphatase levels. They also exhibited strong mechanical properties and effective antibacterial activity against both Gram-positive and Gram-negative bacteria. [139]. Zia et al. developed nanohydroxyapatite particles embedded in CS–carrageenan polyelectrolyte complexes, forming physically cross-linked nanostructures. These composites showed bone-like lattice and tensile properties in simulated body fluid, along with a rough surface that promoted apatite formation. They were also cytocompatible, biodegradable, and supported protein adhesion, making them strong candidates for bone tissue engineering applications [140].

CS’s versatility allows it to be used in various forms like fibers, sponges, and hydrogels for cartilage tissue regeneration. Sadeghi et al. developed a novel scaffold by combining 3D printing and impregnation techniques to produce CS–alginate (Alg) structures enhanced with nanohydroxyapatite. The addition of nanohydroxyapatite improved mechanical strength and significantly enhanced cell adhesion and viability. These scaffolds also exhibited strong antibacterial activity, further boosted by the presence of nanohydroxyapatite [141].

Pitrolino et al. developed a multilayered CS-based scaffold for osteochondral injury repair using a combination of freeze-drying and porogen-leaching techniques. The resulting structure had a tailored pore size (160–275 µm) and incorporated 70% nanohydroxyapatite in the bone-like layer for added strength. The scaffold withstood compression and tensile forces without delamination and supported human stem cell adhesion and growth. Cells showed layer-specific morphology—adherent on the bone layer and spherical on the cartilage layer. The design promoted targeted osteogenic and chondrogenic differentiation, making it a strong candidate for minimally invasive cartilage and bone regeneration [142]. A multifunctional, bioinspired 3D scaffold with potential for vascularized bone tissue regeneration was developed using CS, nanohydroxyapatite, and fucoidan via freeze-drying. Incorporation of nanohydroxyapatite into the CS–fucoidan matrix reduced water uptake and retention, enhancing structural stability. In vitro studies using periosteum-derived mesenchymal stem cells confirmed the scaffold’s favorable microstructure for cell adhesion, nutrient exchange, and mineralization. The construct demonstrated excellent biocompatibility and osteogenic potential, indicating its suitability for bone repair and regeneration application [143].

In another study, Gomes et al. developed a nanofibrous scaffold via electrospinning using polycaprolactone, CS, and gelatin derived from cold-water fish skin. The scaffold exhibited favorable characteristics, including high surface porosity, hydrophilicity (as indicated by low water contact angle), mechanical strength, and controlled degradability in both water and cell culture media. The fibrous structure supported strong cell adhesion and proliferation, with cells reaching confluence across all scaffold types, highlighting its potential for soft and hard tissue engineering applications [144].

A bilayered CS–gelatin composite scaffold was recently designed for vascular tissue engineering, demonstrating suitable morphological, mechanical, and biodegradation properties. The porous inner layer supported high surface area, cell adhesion, and fibroblast proliferation, while the outer nonporous layer inhibited external cell infiltration and provided flexibility and elasticity. The scaffold exhibited time-dependent biodegradability, enabling progressive cell infiltration and tissue integration. These characteristics suggest the bilayered construct as a promising candidate for biological blood vessel applications [145].

#### 6.1.7. Wound Healing

CS membranes have been extensively studied for their effectiveness in wound healing, particularly in the treatment of severe burns and injuries. Polyvinyl alcohol–CS composite membranes improve mechanical strength, while glycerol–oleic acid–CS composites enhance biocompatibility and bioabsorbability and enable sustained drug release. CS-based composites play a vital role in wound care by preventing infections and accelerating the healing process. Silver–CS exhibits strong antibacterial properties, while copper–CS and zinc oxide–CS promote wound healing by stimulating collagen deposition, fibroblast proliferation, and re-epithelialization [101].

CS alone has limited adhesiveness in wet environments, leading to the development of catechol-functionalized CS, which forms strong covalent bonds with tissue proteins. To retain CS’s anticoagulant properties, Du et al. created an amphiphilic hydrogel combining hydrophobically modified CS and hydrocaffeic acid-modified CS. This hydrogel showed strong antibacterial activity against *P. aeruginosa* and *S. aureus*, was non-toxic to fibroblast cells, and enabled sutureless wound closure in a rat skin model, demonstrating excellent tissue adhesion and healing potential [146].

While CS is known for its hemostatic properties, its effectiveness is limited in cases of severe hemorrhage or microbial infection [146]. To address CS’s limitations in severe bleeding scenarios, Du et al. developed a micro-channeled alkylated CS sponge using 3D-printed microfiber leaching, freeze-drying, and surface modifications. The sponge rapidly absorbed fluids and restored its shape, outperforming standard gauze, gelatin sponges, and commercial hemostats in rat and pig models of severe liver injury. The micro-channeled alkylated CS sponge also showed strong anti-infective activity against *S. aureus* and *E. coli*, while promoting tissue integration and healing [147].

#### 6.1.8. Drug and Gene Delivery

CS-derived nanoparticles enhance bioavailability, therapeutic efficacy, and sustained drug release while minimizing side effects [148]. They facilitate the formation of peptide-loaded nanoparticles with improved stability and controlled release properties [149]. Modified CNPs have also been shown to enhance insulin delivery by protecting it from gastrointestinal degradation and improving absorption [150,151,152].

Yu et al. developed CNPs modified with octenyl succinic anhydride to enhance the anti-inflammatory and antioxidant effects of two model drugs, quercetin and curcumin. These nanoparticles demonstrated pH-sensitive drug release, with a more rapid release occurring at approximately pH 6.0 [153]. Barbosa et al. developed fucoidan–CS polymeric nanoparticles for quercetin delivery using a polyelectrolyte self-assembly method, with fucoidan/CS weight ratios of 1/1, 3/1, and 5/1. Quercetin loading ranged from 110 ± 3 to 335 ± 4 mg/mL as the fucoidan content increased. The nanoparticles measured 300–400 nm and had surface charges around +30 mV, indicating good stability. While 1 fucoidan–1 CS nanoparticles became unstable with increasing pH, 3 fucoidan–1 CS and 5 fucoidan–1 CS remained stable across pH 2.5–7.4, suggesting suitability for oral delivery. These formulations showed strong antioxidant activity and protected quercetin from degradation, enhancing its absorption [154]. Kheiri et al. developed magnetic core–shell nanogels composed of iron oxide coated with CS and polyacrylic acid using glutaraldehyde for cross-linking to deliver the anticancer drug 5-fluorouracil. In vitro studies simulating both physiological and tumor-like environments showed that the nanogels released more 5-fluorouracil under acidic conditions (pH 4.5), typical of tumor tissues, compared to neutral pH (7.4), representing normal body fluids. The drug release followed a Fickian diffusion mechanism, indicating it was primarily controlled by the drug’s diffusion through the nanogel matrix [155]. As non-viral vectors, CNPs offer superior transfection efficiency and effective gene protection [156,157,158]. Methoxy polyethylene glycol-modified trimethyl CS doxorubicin nanoparticles, which incorporate methoxy polyethylene glycol-modified trimethyl CS, doxorubicin, and cis-itaconic anhydride, demonstrate stronger antitumor activity compared to doxorubicin or plasmid DNA alone [159,160]. Additionally, O-carboxymethyl CNPs have been found to inhibit tumor migration, while poly-β-amino ester nanoparticles conjugated with thiolated O-CMCS achieve higher transfection rates [161,162]. Further modifications with targeted ligands enhance tumor-specific gene delivery, increasing therapeutic potential [163].

Arami et al. developed iron oxide-based nanoparticles coated with polyethylene glycol, lactobionic acid, CS, and polyethylenimine to create a positively charged carrier capable of binding small interfering RNA. These nanoparticles successfully delivered survivin-targeting small interfering RNA to human breast cancer cells and leukemia cells in vitro. The nanoparticles effectively complexed with the genetic material, had a suitably small size for gene delivery, and demonstrated biocompatibility without causing toxicity to healthy cells [164].

Zhao et al. developed a targeted small interfering RNA delivery system using CNPs modified with HA to target CD44 receptors on activated macrophages. These nanoparticles were embedded into poly(lactic-co-glycolic acid) and poly(cyclohexane-1,4-diylacetone dimethylene ketal) microparticles, forming a composite system for rheumatoid arthritis treatment. The CS’s positive charge improved small interfering RNA distribution and protection. The system achieved 70% sustained release over 15 days, with in vivo results showing stable blood levels and effective therapeutic response for up to 8 days [165].

### 6.2. Applications in Food and Environmental Science

#### 6.2.1. Food Packaging and Preservation

CS and its derivatives exhibit excellent film-forming and antibacterial properties, making them highly suitable for food packaging and preservation. CS films are non-toxic, edible, and water-insoluble, providing superior antiseptic properties, freshness retention, and moisture control compared to conventional plastic wrap [166,167]. Additionally, they are biodegradable, helping to reduce environmental pollution. Uranga et al. developed a citric acid–fish gelatin–CS composite film with strong UV-blocking capabilities, where CS effectively inhibited the growth of *E. coli* [168].

#### 6.2.2. Food Industry Additives

CS and its derivatives are widely utilized in the food industry as thickeners, decolorants, and stabilizers. They effectively absorb food pigments, forming non-absorbable complexes that mitigate pigment toxicity. As a cationic flocculant, CS facilitates the aggregation of colloidal particles, chelates metal ions, and efficiently adsorbs polyphenolic compounds, thereby reducing the total solids and heavy metal content in liquids. Its antibacterial and antioxidant properties further contribute to preventing lipid oxidation, inhibiting microbial growth, and extending food shelf life. For example, CMCS has been shown to reduce protein and polyphenol content in blackberry juice, helping to prevent turbidity and secondary precipitation during storage [169].

#### 6.2.3. Wastewater Treatment

The contamination of food wastewater, particularly from non-biodegradable dye pollutants, has become an increasing environmental concern. Due to their high toxicity, chemical resistance, and slow degradation, dye pollutants are challenging to remove and treat effectively [170]. CS hydrogel serves as an efficient dye adsorbent, offering advantages such as low energy consumption, ease of operation, high flexibility, biodegradability, and strong adsorption capacity [171,172]. Its excellent swelling ability facilitates water and dye transport, enhancing interactions with binding sites for improved adsorption. The abundant -OH and -NH_2_ groups in CS enable effective physical adsorption of food dyes [173]. Additionally, in acidic aqueous solutions, CS exhibits behavior similar to that of cationic polymers, allowing it to effectively bind with anionic contaminants in wastewater, thereby enhancing the removal efficiency of food dyes [174].

### 6.3. Plant Protection and Growth Enhancement

CS film coatings on seeds inhibit fungal pathogens and enhance plant disease resistance. Chandra et al. reported that CS induced immunity in camellia against vesicular fusarium wilt [175]. Additionally, CS strengthens plant resistance to disease, lodging, and cold stress while reducing the need for pesticides and fertilizers, thereby minimizing soil pollution. It also binds heavy metals, contributing to soil purification, and serves as a seed treatment, fertilizer buffer, and pesticide additive [176]. Furthermore, CS improves pest control by regulating pesticide release, prolonging its effectiveness [177].

To provide a clearer overview of the versatility of CS, Table 3 summarizes its main application areas along with specific examples and associated functional benefits.

## 7. Chitosan as a Gel-Forming Agent

CS is a unique biodegradable biopolymer with low toxicity and a positively charged structure at acidic pH and consequently has been widely investigated for gel applications. Its ability to undergo sol-to-gel transitions makes it particularly valuable in biomedical fields [178,179,180]. Unlike naturally occurring gel-forming polysaccharides such as alginates and carrageenans, CS does not exhibit inherent gelation properties, necessitating innovative approaches to induce gel formation [181]. CS hydrogels are typically formed through two primary mechanisms: physical cross-linking or chemical cross-linking.

### 7.1. Physical Gelation

The physical gelation of CS is primarily influenced by its pKa, DD, and molecular weight, all of which affect its solubility and responsiveness to pH changes [182]. As a polyelectrolyte, CS interacts with oppositely charged molecules within a pH range of 4–6, facilitating self-assembly. This process occurs rapidly, but can be modulated by adjusting ionic strength or cross-linker concentration. While physical gels offer tunable swelling and controlled degradation, they generally exhibit lower mechanical strength compared to chemically cross-linked counterparts [183].

#### 7.1.1. Controlled N-Acylation Gelation

Moore et al. studied CS gelation behavior in solutions with acyl anhydrides, identifying the key factors: CS and anhydride concentrations, temperature, and molecular weight. Gelation took place through N-acylation, decreasing the polymer solubility. The gelation was accelerated by higher temperatures and increased CS concentrations, while larger acyl anhydride molecules lowered it due to steric hindrance. After gelation, N-acylation continued, causing gel compaction and water loss. A minimum acetylation degree (~80%) was essential for gel formation and specific glucan residue distribution [184]. Domard et al. confirmed that a minimum molar ratio (R ≥ 1.3) and polymerization degree (DPw ≥ 280) are necessary for macroscopic gel formation. Ethanol showed better gelation properties compared to 1,2-propanediol [185]. Several conclusions of these studies are detailed in Table 4.

#### 7.1.2. Base-Induced Gelation

CS hydrogels have been successfully formed without external cross-linkers using aqueous NaOH or gaseous NH_3_. The mechanical properties of hydrogels derived from high-molecular-weight CS (≈640,000 g/mol) at polymer concentrations ≥2.0% were enhanced due to increased chain entanglement. Their physicochemical and mechanical characteristics can be fine-tuned by adjusting polymer concentration and gelation conditions.

CS (1.5%) hydrogels were used for cardiac therapy (myocardial infarction regeneration), underscoring a successful integration onto the epicardial surface, partial degradation, and cell infiltration [186]. A CS hydrogel whose fragments ranged from 20 µm to 150 µm with different deacetylation (DA) degrees was tested in a spinal cord injury rat model. It promoted tissue regeneration, reduced scarring, supported axon regrowth, and improved locomotor recovery [187]. CS hydroalcoholic solutions are gellified while neutralized with alkaline compounds such as NaOH or NH_4_OH, resulting in varying stiffness correlated with the DA. Highly deacetylated gels improved cell adhesion, while low-acetylation gels showed better tissue regeneration and neovascularization. Highly acetylated CS hydrogels were softer, degraded quickly in vivo, and were less effective for stem cell applications [188]. Articles where the base-induced gelation was considered in the case of CS are summarized in Table 5.

#### 7.1.3. Ionic (Ionotropic) Gelation

Sacco et al. [189] developed cylindrical CS hydrogels via a controlled external gelation method using tripolyphosphate (TPP) as a cross-linker. TPP, a multivalent anion, interacts with protonated CS in acidic solution to induce gelation. By adjusting CS, TPP, and NaCl concentrations, the mechanical strength and homogeneity of the hydrogels were optimized. TPP showed stronger binding to CS than pyrophosphate (PPi), leading to more uniform and stable gels. Circular dichroism indicated that gelation involves CS chain reconfiguration. PPi-based gels formed through a distinct three-step process, resulting in different structural and mechanical properties compared to TPP-based gels [190].

A comparative study of TPP- and PPi-cross-linked CS hydrogels revealed key differences. TPP hydrogels showed higher network homogeneity, greater mechanical stability, and remained intact for up to six weeks, while PPi hydrogels degraded within days. TPP also had higher cross-linking density (0.43 ± 0.01 mm^2^/mm^2^ vs. 0.28 ± 0.02 mm^2^/mm^2^), allowing better control of molecular diffusion and drug release. Pore size analysis confirmed TPP’s suitability for sustained delivery. Both hydrogels were non-toxic, but TPP hydrogels improved cell adhesion and proliferation, supporting their use in tissue engineering and regenerative medicine [191].

A slow diffusion-based method was developed to create macroscopic CS hydrogels embedded with silver nanoparticles stabilized by lactose-modified CS (Chitlac). This technique enabled uniform cross-linking and nanoparticle distribution without localized hardening. UV-vis and transmission electron microscopy confirmed the stability and dispersion of the nanoparticles. The hydrogels showed synergistic antimicrobial activity against *S. aureus*, *E. coli*, *S. epidermidis*, and *P. aeruginosa*, effectively disrupting mature biofilms. Biocompatibility tests on keratinocytes and fibroblasts confirmed non-cytotoxicity, supporting their potential for biomedical applications. [192].

Another study introduced 6-phosphogluconic trisodium salt (6-PG−Na^+^) as a novel, non-toxic cross-linker for chitosan (CS) ionic hydrogels. Formed via ionic interactions between CS and 6-PG−Na^+^, the resulting hydrogels were biocompatible, adhesive, and suitable for topical use. They showed no dermal irritation, high extensibility, and followed first-order drug release kinetics, highlighting their potential for drug delivery and wound dressing applications [193].

Huang et al. conducted extensive research on the formation mechanism of CS–TPP microgels, analyzing their properties by adjusting key factors such as the DA and molecular weight of CS, pH, ionic strength, and the concentrations of both the polymer and cross-linker. Their findings revealed that gelation kinetics could be significantly slowed by simply modulating the amounts of TPP and monovalent salt (NaCl) [183]. The formation rates of CS–TPP micro- and nanogels were highly dependent on NaCl and TPP concentrations, occurring in two distinct stages. At 150 mM NaCl, colloidal stability improved by weakening CS–TPP binding, reducing bridging, and narrowing size distribution. However, at 500 mM, binding was too weak to support gel formation. Given the importance of stability against aggregation and dissolution in biomedical use, Huang et al. further examined the influence of chitosan’s DA and particle concentration under physiological pH and ionic strength conditions [194,195].

Rampino et al. explored the use of cryoprotectants, including trehalose, polyethylene glycol, and mannitol, to enhance nanoparticle stability after drying. Among these, trehalose proved to be the most effective, preserving the structural integrity of the nanoparticles. In vivo biocompatibility tests conducted using chick embryos further confirmed the safety of the optimized formulations [196].

Alshamsan et al. developed indomethacin-loaded CNPs with optimized formulation parameters to improve drug delivery. The nanoparticles remained stable for six months, maintaining structural integrity and charge. They enabled sustained, controlled release of indomethacin, and biocompatibility tests confirmed their safety and potential as effective carriers for targeted drug delivery [197].

Another study developed CNPs using TPP-based ionic cross-linking to co-load aspirin (hydrophilic) and probucol (hydrophobic) for restenosis treatment. Imaging confirmed spherical nanoparticles, with aspirin in amorphous form and probucol in a crystalline state. In vitro studies showed sustained aspirin release over 24 h and extended probucol release up to 120 h, demonstrating effective dual drug delivery [198].

CS–TPP nanogels have also been shown to efficiently encapsulate highly hydrophobic drugs, including antihypertensive agents, with high drug-loading capacity and controlled release properties [199]. Furthermore, these nanogels demonstrated their suitability for encapsulating anticancer drugs such as doxorubicin, further reinforcing their potential in oncology applications [200].

Butyrate, a short-chain fatty acid with anti-inflammatory properties, inhibits reactive oxygen species release, but has a short half-life, limiting its therapeutic efficacy. To enhance its stability and effectiveness, CS–HA nanoparticles (B-NPs) were developed for controlled release, ensuring a sustained inhibitory effect. Unlike free butyrate, B-NPs maintained long-term reactive oxygen species suppression [201].

CS–HA nanogels have been used for protein encapsulation, including VEGF and platelet-derived growth factor BB (PDGF-BB). Parajó et al. developed CS–HA nanoparticles via ionic gelation, using bovine serum albumin and heparin as stabilizers. The system achieved high encapsulation efficiencies, 94% for VEGF and 54% for PDGF-BB. In vitro, VEGF was fully released within 24 h, while PDGF-BB showed sustained release over about one week [202]. These findings highlight the potential of HA-functionalized nanoparticles for controlled drug delivery, leveraging receptor-mediated uptake for improved bioavailability.

Building on this approach, HA-coated CS–TPP nanoparticles were taken up by RAW 264.7 macrophages via CD44-mediated endocytosis, while alginate-coated counterparts used a different, unidentified receptor. Receptor clustering influenced uptake: high-affinity nanoparticles caused stronger clustering, but lower internalization, whereas lower-affinity particles achieved more efficient uptake, enhancing HA-mediated drug delivery [203].

To target tumor-reinitiating cancer stem-like cells, doxorubicin-loaded CS-coated nanoparticles were developed to bind CD44 and release the drug in acidic environments. This approach increased doxorubicin cytotoxicity sixfold in CD44^+^ cells within 3D tumor spheroids and significantly reduced tumor size in an orthotopic xenograft model, with minimal systemic toxicity [204].

Deng et al. further developed CS–HA nanogels designed to overcome drug resistance in breast cancer. These nanogels co-encapsulated doxorubicin, an antineoplastic drug, and miR-34a, a potent endogenous tumor suppressor. In vitro and in vivo studies demonstrated that this formulation enhanced the antitumor effects of both agents, presenting a promising strategy for improving cancer therapy [205].

Alhasan et al. [206] studied HA-coated CS–TPP nanogels and found that HA modification reduced protein adsorption and immunogenicity. Gene Ontology analysis showed that HA–CNPs adsorbed fewer inflammatory proteins and selectively bound anti-inflammatory ones like inter-alpha-trypsin inhibitor heavy chain 4 and alpha-1-acid glycoprotein, unlike CS– and Alg–CNPs, which adsorbed pro-inflammatory clusterin. In CHO-K1 cells, uncoated CNPs reduced viability and increased oxidative stress, while HA–CNPs scavenged reactive oxygen species and minimized cytotoxicity, emphasizing HA’s role in improving biocompatibility [207].

A recent study explored the development of a self-assembled sodium Alg–CS nanogel for tilmicosin delivery to treat *S. aureus*-induced bovine mastitis. The nanogel was formed through physical gelation using Ca^2+^ cross-linking, enabling pH-responsive sustained drug release. This formulation exhibited a high encapsulation efficiency (67.89%), enhanced antibacterial activity, and improved bioavailability compared to conventional tilmicosin formulations. In vivo trials demonstrated a 75% cure rate, significantly outperforming commercial tilmicosin injections, which achieved only a 50% cure rate [208].

Additionally, another study focused on the development of an Alg-coated CS nanogel for the controlled topical delivery of silver sulfadiazine to enhance burn wound healing. The nanogel was prepared via ionotropic gelation using TPP as a cross-linker and was optimized to contain 0.4% sodium Alg and 0.414% sulfadiazine. This formulation exhibited sustained drug release, superior antibacterial activity against *S. aureus* and *P. aeruginosa*, and greater therapeutic efficacy than commercial sulfadiazine creams. In vivo studies confirmed that the optimized nanogel formulation accelerated wound healing and enhanced drug retention, making it a promising alternative for burn treatment [209].

Shafiee et al. developed core–shell CS–Alg nanogels for controlled mupirocin delivery using ionotropic gelation, with TPP and CaCl_2_ as cross-linkers. Two formulations, Alg-coated CS and CS-coated Alg, showed high drug loading and sustained release over 72 h (96% for CS-coated Alg, 70% for Alg-coated CS). Antibacterial tests confirmed efficacy against *S. aureus* and *P. aeruginosa*, and in vitro studies showed high biocompatibility. The positively charged CS-coated Alg nanogel demonstrated superior delivery performance [210].

A novel CS–Alg gelling system was developed using controlled pH reduction via D-glucono-δ-lactone, enabling homogeneous gel formation without calcium. Poly-M or poly-G Alg ionically interacts with CS as pH drops, triggering gelation through CS protonation. Mechanical properties were tunable, higher D-glucono-δ-lactone levels produced stiffer gels, while lower concentrations yielded softer, swellable structures. Poly-M Alg formed stronger gels than poly-G due to better charge alignment. These hydrogels show strong potential for tissue engineering, drug delivery, and scaffold applications [211].

Another study explored the development of CS–κ-carrageenan nanoparticles using ionotropic gelation. The optimized nanoparticles exhibited a reduced size (150–300 nm), a lower zeta potential (+50–60 mV), and enhanced stability for up to nine months. The incorporation of TPP improved production yield (25%–35%) and increased mucoadhesive properties, making these nanoparticles promising carriers for mucosal drug delivery [212]. Additionally, researchers developed CS–carrageenan–TPP nanoparticles via ionotropic gelation for pulmonary and nasal protein delivery. The stable, positively charged particles (~300 nm, +40 mV) showed high biocompatibility, resisted lysozyme degradation, and enabled controlled bovine serum albumin release. Spray-drying with mannitol produced microparticles (~2.3 μm) suitable for inhalation. In vitro studies confirmed no cytotoxicity, no inflammatory response, and maintained epithelial barrier integrity, supporting their potential as mucosal protein carriers [213].

Lerouge et al. investigated an injectable, bioadhesive hydrogel composed of catechol-modified CS cross-linked with sodium bicarbonate for applications in drug delivery and tissue engineering. This hydrogel rapidly gels within five minutes at 37 °C, mimicking the adhesive properties of mussels to ensure strong tissue bonding while maintaining physiological pH and osmolality. It demonstrates high mechanical strength, with a resistance of 90 kPa at 50% strain, and exhibits shear-thinning behavior, facilitating minimally invasive injection. The rapid gelation process also minimizes catechol oxidation, thereby preserving the hydrogel’s structural integrity [214].

A biocompatible hydrogel based on carboxymethyl cellulose and Alg cross-linked via electrostatic and ionic interactions was developed for wound healing. It incorporated epidermal growth factor for enhanced tissue regeneration and featured tunable mechanics, a porous 3D structure (~50–100 µm), pH-responsive swelling, and sustained epidermal growth factor release. The hydrogel showed >97% cell viability, <1% hemolytic activity, and strong antibacterial activity against *S. aureus* and *E. coli*. In vivo, it accelerated re-epithelialization and improved wound healing, supporting its potential for advanced wound care [215].

Mitsuhashi et al. developed a CS-based hydrogel to prevent peritoneal adhesions by cross-linking N-succinyl CS with multivalent metal ions (Al^3+^, Ca^2+^, Fe^3+^). Ionic interactions between CS functional groups and metal ions formed a stable 3D network. N-succinyl CS–Al^3+^ hydrogels showed the best mechanical strength, stability, and controlled degradation, with low cytotoxicity, though some toxicity was attributed to CS’s polycationic membrane-disruptive properties [216].

A recent study developed pH-responsive tanshinone IIA-loaded CS nanogels via ionic gelation with sodium TPP, aiming to enhance antibacterial activity against *S. mutans*. The nanogels showed 91.41% encapsulation efficiency, pH-sensitive release (98% at pH 4.5; 76% at pH 7.4), and improved stability. They increased antibacterial efficacy fourfold and inhibited 72% of biofilm formation at 500 μg/mL, demonstrating strong biofilm penetration and controlled drug release, highlighting their potential as an antimicrobial therapy [217].

Furthermore, a dual ionically cross-linked hydrogel was developed by incorporating CS-functionalized halloysite nanotubes into a poly(acrylamide-co-acrylic acid) network, reinforced through chemical cross-linking and Fe^3+^ coordination. This design significantly improved mechanical properties, achieving high tensile strength (3.06 MPa), stretchability (>2000%), and toughness (47.6 MJ/m^3^) while maintaining ~80% water content. The hydrogel also showed excellent self-recovery—97.9% after 200% strain and 91.5% after 1000% strain [218]. Several articles in which the ionotropic method has been applied for CS are highlighted in Table 6.

#### 7.1.4. Thermally Induced Gelation

Thermosensitive gelling systems are stable, low-viscosity aqueous solutions that transition from a sol to a gel state upon heating, typically at neutral pH, within minutes.

Thermosensitive CS hydrogels, particularly those formulated with glucose-1-phosphate (G1-P), showed promising results for in situ drug delivery. These hydrogels transition to a gel state at body temperature, offering a biocompatible platform for sustained drug release [219]. Different polyol-phosphate agents, such as β-glycerophosphate (β-GP), G1-P, and glucose-6-phosphate (G6-P), influence the gelation speed; consequently, β-GP causes the fastest gelation (~2 min), while G6-P delays it due to stronger hydration layers. The use of higher concentrations of gelling agents and increased CS viscosity can accelerate or decrease the gelation process [220].

A CS–β-GP hydrogel was developed where a demineralized bone matrix for cartilage tissue engineering was integrated, which enhanced the mechanical strength, bioactivity, and stem cell viability. The system stimulated the glycosaminoglycan production for cartilage formation [221]. Additionally, CS hydrogels have been combined with inorganic compounds such as silver (Ag) and silver–palladium (Ag–Pd) nanoparticles to improve antibacterial properties and osteogenic potential for bone tissue engineering [222].

Liang et al. designed a mussel-inspired, injectable CS hydrogel with rapid gelation at 37 °C using electrostatic interactions and thermally induced gelation. This hydrogel showed promising results for wound healing and tissue engineering, combining biodegradability, adhesiveness, and biocompatibility for regenerative medicine [223]. The previously mentioned results are summarized in Table 7.

#### 7.1.5. Electrostatic Interactions

Delair et al. developed a self-assembled CS–dextran sulfate nanogel via electrostatic interactions, showing reversible assembly controlled by salt concentration. The nanogel underscored high colloidal stability, with particles ranging between 350 and 580 nm, and a positively charged surface, making it an excellent choice for drug delivery applications [224].

In another study, a succinoglycan (SG)–CS hydrogel was developed with improved mechanical and thermal properties, showing a pH-responsive drug release and increased biocompatibility. The hydrogel exhibited antibacterial activity against several well-known bacteria, such as *S. aureus* and *E. coli*, making it a promising tool for biomedical uses [225].

A separate study enhanced 3D-printed CS hydrogel scaffolds by reinforcing them with silk particles, improving compressive strength, accuracy, and biodegradability. These scaffolds promoted fibroblast adhesion, showing potential for tissue engineering [226]. A piece of CS-coated cotton fabric was developed using electrostatic cross-linking, significantly improving the following characteristics: antibacterial properties, moisture management, and fabric performance, with strong antibacterial activity [227].

Carrageenan–CS hydrogels developed through ionic gelation exhibited good swelling capacity and mechanical stability, with future potential applications in drug delivery, tissue engineering, and bone regeneration [228].

Hoang et al. obtained a dual-cross-linked CS–Alg hydrogel for pH-responsive oral drug delivery. This matrix controlled the ketoprofen release, maintaining sustained release at neutral pH, with good biocompatibility and biodegradability [229].

Umerska et al. developed cross-linker-free polyelectrolyte complex nanoparticles made from HA and CS for pharmaceutical use. These nanoparticles had tunable sizes and surface charges, influenced by the polymer composition and molecular weight, offering stable drug delivery systems [230].

The impact of DA on nanogel stability and biological effects remains underexplored, though adding Zn^2+^ ions can improve the stability of CS–HA polyelectrolyte complexes [231].

Zucca et al. explored the development of chondroitin sulfate–CS nanogels where a complex that consisted of naringenin-β-cyclodextrin was incorporated for the potential treatment of retinopathy related to diabetes. Multiple formulations were developed and characterized considering the polymer concentration, ratio of chitosan, pH, and particle size. The nanogels were assessed for cytotoxicity and internalization in human umbilical vein endothelial cells, showing promising results in diabetic retinopathy, delivering effectively the active complex [232].

Ali et al. highlighted the importance of electrostatic interactions between carrageenan and CS in the development of nano-in-micro hydrogels. The interactions favored the encapsulation of curcumin rhamnosomes in the microbeads, facilitating controlled release and enhancing the antimicrobial properties [233]. Several articles in which the CS gels were prepared through electrostatic interactions are outlined in Table 8.

#### 7.1.6. Hydrogen Bonding Interactions

Wang et al. used sodium alginate–CMCS polymers as gel-forming agents, developing hydrogel beads through physical cross-linking, relying on hydrogen bonding and electrostatic interactions in a citric acid solution. These beads showed increased swelling capacity, good biocompatibility, and strong potential for drug delivery, offering a non-toxic biomedical alternative [234].

Cui et al. designed an injectable hydrogel based on CS that formed a gel at room temperature and reverted to a solution at 80 °C. This thermoreversible and pH-responsive hydrogel also had self-healing capabilities, making it ideal for gastrointestinal drug delivery and controlled therapeutic release [235].

Nystatin was incorporated in a CS hydrogel for sustained release for the treatment of fungal infections, showing that physically cross-linked hydrogels had faster drug release and greater absorption under acidic conditions. Chemically cross-linked hydrogels provided more controlled, prolonged release. Physically cross-linked hydrogels exhibited the highest antifungal efficacy against *Candida* species [236].

Another study introduced a tetraethylenepentamine (TEPA)-cross-linked CS oligosaccharide hydrogel for removing hexavalent chromium (Cr(VI)) from water. The hydrogel outlined a high adsorption capacity (148.1 mg/g), rapid removal kinetics, and excellent reusability, making it a cost-effective and sustainable solution for water purification [237].

The preparation method of hydrogen bonding interaction was used in several articles where CS was used as a gelling agent (Table 9).

#### 7.1.7. Non-Covalent Chitosan Systems

Garcia et al. evaluated the mechanical and rheological properties of Pluronic F127 thermosensitive hydrogels loaded with CS and grafted with HA and propolis to assay the effectiveness in dermatitis. The development of the final formulation consisted in the poloxamer gel being developed, followed by the incorporation of the CS and HA, and at the end, propolis was added. The results suggest that these composite hydrogels exhibit good mechanical strength and rheological properties, with several correlations being noted regarding these evaluated parameters, and it is expected that in the future, the developed formulations will be applied to wounded skin [238].

In another study conducted by Abdollahi et al., Alg-tetracycline beads were incorporated in a CS–Pluronic–agarose hydrogel that served as a dressing for skin wound treatment. This procedure provided a controlled and sustained release of the active ingredient, a well-known drug used to treat bacterial infections with common pathogens, underscoring its increased potential for infection treatment. Considering the results obtained, the developed system showed good results in domains where an antibacterial-controlled-drug release is considered [239].

Su et al. developed a hydrogel for which CS and agarose were used as gel-forming agents integrating several therapeutic strategies: chemotherapy, photodynamic therapy, and photothermal therapy to effectively combat bacterial infections. The combination of the previously mentioned therapies significantly improved the hydrogels’ ability to eradicate the infections, offering a multifaceted approach that addresses bacterial infections [240].

### 7.2. Chemical Gelation

**Figure 4:**
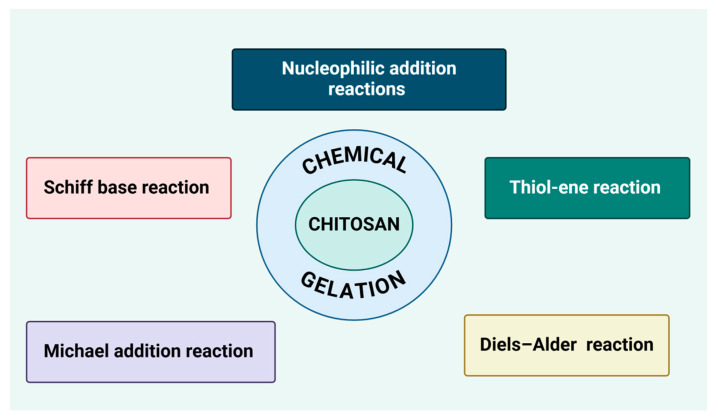
Chemical reactions of CS. Created with BioRender (web application, accessed February 2025; BioRender Inc., Toronto, ON, Canada; www.biorender.com). Figure prepared by Pușcașu C.

The chemical cross-linking (Figure 4) of hydrogels involves the formation of covalent bonds between polymer chains, leading to the development of a three-dimensional network that enhances the gel’s stability, rigidity, and durability. CS, which contains reactive -NH_2_ and -OH groups, readily interacts with chemical cross-linking agents, allowing for improved mechanical properties while maintaining its bacteriostatic activity to inhibit bacterial growth. This cross-linking technology is widely utilized in bioengineering and the fabrication of biomaterials for medical applications [241], including wound dressings, controlled drug delivery systems, and other biomedical uses [242]. For instance, cross-linked hydrogels incorporating CS, glutaraldehyde, and polyvinyl alcohol have demonstrated significant potential in biotechnology applications, further expanding their functional versatility.

The Schiff base reaction holds significant potential for the formation of three-dimensional lattice structures through cross-reactions that establish strong cross-linking connections between polymer molecules. This method, widely employed in polysaccharide-based hydrogel synthesis, involves the introduction of carbonyl groups into a modified polysaccharide backbone, which subsequently reacts with amino groups in proteins to form covalent imine bonds known as Schiff bases. This approach enhances the structural integrity and functionality of hydrogels, making it particularly valuable for biomedical applications, including drug delivery, tissue engineering, and wound healing [241,243,244].

The Schiff base reaction, in which a primary amino group reacts with an aldehyde, is a widely used method for obtaining stable and functional structures. Among chemical cross-linkers, glutaraldehyde is frequently employed due to its high efficiency; however, its toxicity poses significant limitations. In contrast, diethyl tartrate offers superior biocompatibility, reducing adverse effects on biological systems. Additionally, polysaccharides such as galactomannan and maltodextrins can facilitate the cross-linking of CS by undergoing periodate oxidation to introduce aldehyde groups, enhancing the formation of Schiff bases. Genipin, a naturally derived cross-linker, presents a less toxic alternative to glutaraldehyde, making it more suitable for biomedical applications. However, its use is not without challenges, as it can react with encapsulated drugs and exhibits a tendency toward self-curing, which may affect the long-term stability of hydrogels [245].

CS-based hydrogels undergo Schiff base reactions, resulting in enhanced mechanical strength, bioactivity, and the ability to provide controlled drug release [242,246]. A notable example is the development of CS-PC–A2–RA hydrogels, synthesized through cross-linking with 2,3,4-trihydroxybenzaldehyde and subsequent modification with pectin, bioactive glass, and rosmarinic acid. The three -OH groups in 2,3,4-trihydroxybenzaldehyde introduced additional binding sites under acidic conditions, while its aldehyde groups formed imine bonds with the amino groups of the CS chain. Electrostatic interactions between pectin and CS facilitated the formation of polyelectrolyte complexes, while positively charged hydrogen atoms in CS bonded with negatively charged oxygen atoms of rosmarinic acid. Furthermore, calcium ions from bioactive glass interacted electrostatically with carbonyl groups in pectin, generating multiple synergistic interactions. Notably, Schiff base reactions contributed more significantly to the structural integrity of the hydrogel than ionic interactions and hydrogen bonding. The resulting hydrogels exhibited excellent mechanical properties, strong antioxidant activity, and cytotoxic potential, with the ability to suppress cancer cell proliferation [247].

In most covalent bonds, a considerable amount of energy is required to break the bond between atoms. However, Schiff bases form transient (dynamic) covalent bonds that can naturally dissociate and reassociate or respond to external stimuli, enabling self-healing properties and biodegradability within their three-dimensional network [248]. Zhou et al. [249] reported that Schiff base hydrogels demonstrate self-healing behavior and enhanced injectability due to the reversible nature of Schiff base formation, particularly in aqueous environments. These materials also exhibit pH sensitivity, undergoing accelerated hydrolysis in acidic conditions. Expanding on this concept, Guo et al. developed a novel hydrogel for wound dressing applications, specifically designed for chronic wound healing. This hydrogel was synthesized through a Schiff base reaction between oxidized HA and CMCS, demonstrating promising biomedical potential due to its biocompatibility, biodegradability, and capacity for controlled degradation [250].

Ding et al. synthesized a hydrogel based on two biopolymers, acrylamide-modified CS and oxidized Alg, utilizing covalent cross-linking to form imine bonds. Their study revealed that the mechanical properties and self-healing behavior of the hydrogels were significantly influenced by cross-linking duration and pH conditions [251]. Ma et al. developed an injectable hydrogel by incorporating hybrid nanoparticles of oxidized alginate and hydroxyapatite with CMCS, also forming imine bonds. Rheological tests demonstrated that the storage modulus increased with both higher oxidized alginate concentrations and extended oxidation times, indicating that these hydrogels have strong potential for bone tissue engineering applications [252,253].

Zhang et al. employed a Schiff base reaction to synthesize hydrogels from oxidized CS and zinc oxide, assessing their biocompatibility with multiple cell lines, including 293T cells, blood cells, and 3T3 cells. The hydrogels exhibited favorable biocompatibility, high swelling capacity, and porous structures, along with potent antibacterial activity against *B. subtilis*, *C. albicans*, and *S. aureus*. Furthermore, in vivo experiments demonstrated their ability to accelerate wound healing in a rat scald model [254]. In another study, Oh et al. fabricated hydrogels via a Schiff base reaction involving gelatin, oxidized sodium Alg, CS, and salicylic acid. These hydrogels exhibited remarkable wound healing properties while demonstrating no observable toxicity, further supporting their potential for biomedical applications [255].

With the growing emphasis on green chemistry, there is increasing interest in chemical reactions that minimize the use of hazardous solvents, reduce reaction time, and enable the formation of well-controlled structures under mild conditions [256].

The Diels–Alder reaction, first described by Otto Diels and Kurt Alder in 1928, aligns with these principles as a thermoreversible process [257]. The Diels–Alder reaction proceeds under mild conditions in aqueous environments, does not require a catalyst, and generates no by-products, making it a highly sustainable and efficient cross-linking approach [258].

Li D et al. developed hybrid pectin–CS hydrogels [242], synthesized by combining furfural-modified pectin with maleimide-modified CS through the Diels–Alder reaction, based on the Brønsted–Lowry acid-base theory. Since both pectin and CS are amphoteric compounds, their ionization under different pH conditions influences the hydrogel properties. In an acidic environment, the -COOH groups in pectin and -NH_2_ groups in CS exist as -COOH_2_^+^ and -NH_3_^+^, respectively, while at pH levels above 7, they deprotonate to form -COO^−^ and -NH_2_. Furfural modification of pectin introduced conjugated diene fractions, whereas grafting 6-maleimidohexanoic acid onto CS produced protodiene fractions. While earlier studies indicated that reversible Diels–Alder reactions typically required high temperatures (above 100 °C) [259], rendering them unsuitable for physiological conditions, recent findings demonstrate that furan and maleimide can undergo a reversible Diels–Alder reaction at 37 °C, making this approach viable for tissue engineering applications.

The Michael addition reaction is a conjugate addition process in which a nucleophilic reagent reacts with an α, β-unsaturated compound containing one or more carbon–carbon double bonds attached to functional groups. Its broad applicability lies in its ability to introduce diverse functional groups into conjugated systems, enabling the construction of complex molecular architectures and the development of novel chemical structures with potential applications across various scientific disciplines. Guar Esti et al. successfully synthesized water-soluble CS derivatives through the sulfurylation of base polymers using thiolactic acid. Subsequently, a pH-sensitive CS-based hydrogel was fabricated via a sulfhydryl–Michael addition reaction involving the covalent cross-linking of sulfhydryl-modified CS with water-soluble bis-maleimide. The resulting hydrogels exhibited excellent mechanical properties, structural stability, in vivo degradability, and thermal stability, highlighting their potential for biomedical applications [242].

The thiol-ene reaction involves the addition of a thiol (-SH) group to a carbon–carbon double bond of an olefin, resulting in the formation of a new carbon–sulfur single bond. This versatile reaction is widely utilized in polymer modification, enabling the development of functional materials and bioactive compounds. By incorporating sulfur-containing groups into polymer structures, the thiol-ene reaction enhances polymer adaptability and functionality, facilitating advancements in materials science and biotechnology. Li R. et al. applied the thiol-ene reaction to synthesize hydrogels by cross-linking CS thioglycolate-coated liposomes with CS-maleate-coated liposomes. The resulting hydrogels exhibited a porous structure, with variations in the n-SH/n-CS ratio influencing gelation time and swelling behavior, demonstrating their potential for tunable biomedical applications [242].

Nucleophilic addition reactions occur when nucleophilic reactants, such as alcohols, amines, and thiols, interact with electrophilic regions in the reactants, leading to the disruption of ring structures and the formation of new chemical bonds. This process facilitates the synthesis of novel compounds or the expansion of existing ring structures, broadening their functional applications [242]. Wang C et al. successfully synthesized 2-N,6-O-sulfated CS (2-N,6-O-SCS) by modifying CS with formamide, formic acid, chlorosulfonic acid, and N,N-dimethylformamide. The modified CS was then integrated into gelatin sponges to create 2-N,6-O-SCS-encapsulated scaffolds. These scaffolds were evaluated for their ability to capture VEGF, functioning as an effective cytokine reservoir, demonstrating potential applications in tissue engineering and regenerative medicine [242].

The introduction of the cross-linking structure of the disulfide bond network into the CS coating weakens the hydrogen bonds between the CS macromolecules, causing the macromolecular chains to be more prone to relative motion when subjected to external forces, ultimately resulting in the flexibility of the coating. Modified CS becomes more suitable for antibacterial modification. In the study by Wang et al., thioctic acid was grafted onto CS to alter its properties. With the introduction of disulfide bonds, a new bond emerged, known as disulfide cross-linking. Using the dehydration condensation reaction between the carboxyl groups on the thioctic acid and the amino groups on the CS, a CS molecule containing disulfide bonds, thioctic acid–CS, was obtained [260].

Borate bonds are formed through the reversible condensation of boric acid or its derivatives with diols or phenols, allowing these bonds to dissociate in acidic conditions and impart pH-responsive properties to the resulting materials [261,262]. Due to their dynamic and reversible nature, hydrogels incorporating borate bonds also exhibit self-healing capabilities [263].

Yu et al. developed a pH-responsive hydrogel designed for postoperative tumor recurrence prevention and wound infection control, utilizing CS as the primary backbone. The -NH_2_ groups on CS were coupled with the carboxyl groups -COOH of 3-carboxyphenylboric acid through an amidation reaction to form CS–boric acid. This CS–boric acid was subsequently reacted with diols present in PVA to establish reversible dynamic boronic ester bonds, enhancing the hydrogel’s adaptability and functionality for biomedical applications [264].

The defining characteristics of chemical hydrogels include covalent bonding, which requires chemical modification of CS, leading to the formation of irreversible networks. The amide and ester bond, as well as the Schiff base, are examples of bonds that can occur during the production of chemical hydrogel [265].

## 8. Conclusions

CS-based hydrogels have been extensively explored due to their biocompatibility, biodegradability, and versatility in biomedical and pharmaceutical applications. The present review builds upon existing research by examining various cross-linking methods, gelation mechanisms, and their impact on hydrogel properties. The findings align with previous studies that have demonstrated the tunability of CS hydrogels through physical, ionic, and chemical cross-linking approaches, with each method offering distinct advantages in terms of mechanical strength, swelling behavior, and bioactivity.

The present review highlights recent advances such as Schiff base chemistry, Diels–Alder reactions, and Michael addition, which provide innovative strategies for enhancing hydrogel stability, self-healing capabilities, and pH responsiveness. These findings are consistent with prior research, indicating that dynamic covalent interactions and reversible bonding mechanisms can significantly improve hydrogel adaptability and functionality. Furthermore, the incorporation of nanomaterials and bioactive compounds has been shown to enhance the structural integrity and biological performance of these materials, supporting their potential for drug delivery, wound healing, and tissue engineering applications.

Despite these advancements, several challenges remain. One limitation is the need for more standardized protocols to ensure the reproducibility and scalability of CS-based hydrogel formulations. Additionally, while chemical modifications improve mechanical properties and drug-release kinetics, they may introduce cytotoxicity concerns, requiring further biocompatibility assessments. Another key consideration is the long-term stability of these hydrogels in physiological conditions, as degradation rates must be carefully optimized to match clinical needs. In this context, non-covalent CS-based systems have gained increasing attention for their ability to form biocompatible hydrogels without the use of potentially toxic chemical cross-linkers. Several recent studies have demonstrated the successful use of ionic or physically blended systems incorporating components like Pluronic F127, hyaluronic acid, alginate, and agarose combined with therapeutic agents such as propolis or tetracycline. These non-covalent hydrogels exhibit promising mechanical strength, controlled drug release, and multifunctional therapeutic action, particularly in wound healing and antibacterial applications.

Future research should focus on refining environmentally friendly synthesis methods, optimizing cross-linking strategies to balance mechanical performance and biodegradability, and exploring multifunctional hydrogels that integrate stimuli-responsive behaviors. Moreover, the translation of these materials from laboratory studies to clinical applications requires further investigation into regulatory compliance, large-scale production, and in vivo efficacy. Expanding the application of CS hydrogels in regenerative medicine, biosensing, and antimicrobial coatings presents exciting opportunities for future studies.

## Figures and Tables

**Table 1:** Chitin yield from various biological sources for CS production.

Source	Yield (%) of Chitin	References
Shrimp shells (shrimp waste)	33.37%–36.43% (depending on particle size of shrimp waste)	[52]
Shrimp shells (*P. longirostris*)	26.98%	[53]
Shrimp shells (*P. durarum*)	23.72%	[54]
Crabs’ shells	16.73%	[54]
Fungi (*S. cerevisiae*)	2%	[55]
Fungi *(R. arrhizus)*	8.3%	[56]
Fungi (*C. elegans)*	6.6%	[57]
Fungi (*A. terreus)*	34%	[58]
Insect cuticles (cicada sloughs)	36.6%	[59]
Insects (*T. molitor*)	17.32%	[60]
Insects *(H. parallela)*	15%	[61]
Insects (*B. mori*)	2.59%–20%	[62,63]
Insects (*E. kuehniella*)	9.5%–10.5%	[64]
Insects (*A. pandora*)	22%	[65]
Fungi (*M. domestica*)	8.02%	[66]

**Table 2:** Comparative analysis of chitosan extraction methods based on environmental impact, scalability, efficiency, and other relevant criteria [68,69,70,71,72,73,74,75,78,84,86,87,88].

Extraction Method	Advantages	Limitations	Environmental Impact	Scalability
Physical	Enhances reactivity; reduces processing time when combined with other methods	Requires high energy input; may alter chitin structure	Moderate to high (depending on energy source)	Moderate
Chemical	High yield and efficiency; widely used industrially	Uses harsh chemicals; produces hazardous waste; random deacetylation	High (due to chemical usage and waste)	High
Biological	Eco-friendly; selective deacetylation; high-quality chitosan	Slow process; enzyme cost; sensitive to process conditions	Low	Low to moderate
Green (e.g., supercritical fluids, ionic liquids)	Environmentally sustainable; minimal hazardous waste; high purity	Requires specialized equipment; high initial cost	Low	Currently low to moderate; promising for scale-up

**Table 3:** Main applications of CS and their key features.

Application Area	Specific Examples	Key Benefits
Biomedical	Wound dressings, hemostatic agents, tissue engineering scaffolds, drug/gene delivery	Biocompatibility, biodegradability, mucoadhesiveness, controlled release
Pharmaceutical	Nanoparticles for oral, ocular, and parenteral drug delivery; vaccine adjuvants	Enhances bioavailability, sustained release, low toxicity
Food industry	Edible films/coatings, preservatives, fat replacers	Antimicrobial, antioxidant, non-toxic, extends shelf life
Environmental	Water purification, dye adsorption, heavy metal removal	Chelating ability, biodegradability, flocculation, low cost
Agriculture	Biopesticides, plant growth promoters, seed coatings	Enhances resistance, reduces chemical pesticide use, and promotes sustainable farming
Cosmetics	Moisturizers, antiaging creams, shampoos	Film-forming, moisturizing, antimicrobial, stabilizer

**Table 4:** Preparation of CS hydrogels through controlled re-N-acylation gelation method.

Main Components	Characteristics	Reference
CS, acyl anhydrides (acetic, propionic, butyric, valeric, hexanoic), aqueous acetic acid, methanol	Non-reversible gelation. Gelation time varies with acyl anhydride: acetic (~7 min, fast), hexanoic (~33 min, moderate and stable), and decanoic (~260 min, slow due to steric hindrance). Higher CS concentration (≥2.5% *w*/*v*) accelerates gelation through increased polymer chain interactions. Exhibits syneresis (water loss over time), more pronounced in acetic anhydride gels. Gelation is temperature-dependent, with higher temperatures (30–45 °C) accelerating N-acylation. Stable at pH ~5.0–5.5, but degrades in alkaline conditions.	[184]
CS, acyl anhydrides (acetic, propionic, butyric, valeric, hexanoic), aqueous acetic acid, ethanol, 1,2-propanediol	Minimum DA required for gelation: ~80%. Gelation is temperature-sensitive, occurring faster at higher temperatures (100 °C) due to increased molecular mobility and hydrogen bonding. Post-gelation syneresis (~50% water loss in 21 h). Gels remain stable at pH ~5.0–5.5, but degrade in alkaline conditions. The molecular weight of CS affects gel formation: low-molecular-weight CS fails to form stable gels. Alcohol choice (ethanol vs. propanediol) affects gelation speed, but not the final gel properties.	[185]

CS—chitosan; DA—degree of acetylation.

**Table 5:** Preparation of CS gels through base-induced gelation.

Main Components	Characteristics	Reference
CS, NaOH	Forms hydrophilic hydrogels. Higher CS concentration (>2.0%) results in stronger gels. Swelling and syneresis were observed, particularly at lower CS concentrations. Storage in water leads to gradual hydrogel degradation over time.	[186]
CS, NH_3_	Gelation occurs via NH_3_ diffusion, forming bilayer hydrogels with a soft inner core and denser outer layer. Longer NH_3_ exposure increases stiffness due to deeper -NH_2_ penetration. Heterogeneous structure compared to NaOH-induced gels. Hydrophilic with swelling capacity. More controlled degradation rate than NaOH-induced gels.	
CS, NH_3_, acetic acid, deionized water	Final hydrogel contains only CS and water, closely matching the initial solution concentration (2.5% *w*/*w*). High-molecular-weight CS (Mw ~550 kg/mol, DA ~4 %) enables hydrogel formation at low polymer concentrations (≥1% *w*/*w*). Hydrogel storage modulus at equilibrium: ~1300 Pa.	
CS, NH_3_, acetic acid, 1,2-propanediol	Softer hydrogel, lower stiffness, stable structure. Faster degradation promotes cell infiltration and tissue remodeling. High-DA hydrogels (35%, 20%, 5%) degrade quickly, enhancing tissue remodeling. Promotes angiogenesis with vessel penetration.	[187]
CS, NaOH, acetic acid, 1,2-propanediol	High stiffness, dense structure, and slow degradation. Reduced cell infiltration forms fibrous shells in vivo. Compact structure maintains volume over time. Minimal angiogenesis, vessel growth remains around the hydrogel. High-DA hydrogels (5%, 20%) result in stronger gel formation.	[188]

CS—chitosan, DA—degree of acetylation; Mw—molecular weight.

**Table 6:** Preparation of CS gels through the ionotropic gelation method.

Main Components	Cross-Linker	Characteristics	Reference
CS, acetic acid, TPP, NaCl, glycerol	TPP	Higher TPP concentration leads to increased cross-linking density. NaCl slows down gelation, enhancing uniformity. Mechanical properties vary with CS and TPP concentration: shear modulus ranges from ~9 to 17 kPa. Hydrogels remain stable in PBS with ~20% deswelling over 24 h. Cytocompatibility with NIH-3T3 fibroblast cells, showing no significant cytotoxicity.	[189]
CS, acetic acid, TPP or PPi, NaCl	TPP or PPi	TPP leads to homogeneous, stiffer hydrogels with smaller mesh sizes (10.5–14 nm). PPi forms softer, less homogeneous hydrogels with a larger mesh size (14.7–22.3 nm). TPP-based gels exhibit stronger mechanical properties (higher cross-link density, including Young’s modulus: ~3.8 kPa, higher compressive strength and elasticity). PPi gels degrade faster and have a lower stiffness.	[190]
CS, TPP or PPi, glycerol, acetic acid, NaCl	TPP or PPi	TPP-cross-linked gels exhibit higher connectivity and uniform pore distribution compared to PPi gels. Microscopic TEM analysis revealed TPP gels have a denser, interconnected polymeric network, while PPi gels are more inhomogeneous. TPP gels demonstrated limited degradation in simulated physiological media for up to 6 weeks, while PPi gels degraded rapidly within days. Cytocompatibility studies confirmed both gel types as non-toxic, supporting cell adhesion and metabolic activity. Suitable as biomaterial scaffolds for tissue engineering and controlled drug delivery applications.	[191]
CS, TPP, Chitlac–Ag, glycerol	TPP	3D polymeric network with ionic cross-linking between CS and TPP. Freeze-dried into a flexible, porous membrane for biomedical applications. Hydrophilic with high water absorption (~191% in 48 h), maintaining structural integrity. Antibacterial properties due to embedded AgNPs, effective against *E. coli*, *S. aureus*, *S. epidermidis*, and *P. aeruginosa*. Disrupts mature biofilms, particularly *S. aureus*. Stable in physiological pH (7.4) and 37 °C, but sensitive to extreme pH. Non-toxic to fibroblasts and keratinocytes, supporting biocompatibility.	[192]
CS, 6-PG−Na^+^, acetic acid, NaCl	6-PG−Na^+^	Rapid gelation process, leading to homogeneous and stable hydrogel formation. Controlled drug release, following first-order kinetics. Swelling properties depend on pH: Stable at neutral pH, degrades in acidic conditions (pH ≤ 4.5). Higher cross-linking reduces swelling but enhances structural stability. Forms an adhesive polymeric film, improving wound healing applications. Biocompatible with no cytotoxic effects Potential applications: wound dressings, controlled drug delivery, regenerative medicine.	[193]
CS, TPP, acetic acid, NaCl, deionized water	TPP	Higher TPP concentration accelerates gelation, while NaCl slows it down. Increasing NaCl concentration (0–150 mM) significantly delays micro- and nanogel formation. Lower TPP: glucosamine ratios (<0.03:1) prevent gelation, while higher ratios promote strong exothermic CS–TPP binding. Two-step formation process: (1) primary nanoparticles (~20–50 nm) form first, (2) secondary aggregation leads to larger microgels (~100–200 nm). Microgels continue aggregating over several days, reaching stable sizes (~7 days). CS–TPP binding heat measured via ITC, showing strong cooperative binding at higher TPP levels.	[183]
CS, TPP, NaCl, glucosamine	TPP	Nanogel formation depends on the TPP:CS ratio; excess CS monomer (glucosamine) sites reduce particle size, while excess TPP leads to aggregation and precipitation. Highly sensitive to NaCl concentration, showing a sharp power-law decrease with increasing monovalent salt levels. Effect of pH on gelation: Higher pH enhances TPP binding, accelerating aggregation. CS DD influences stability: Lower DD leads to smaller, more uniform nanogels, whereas higher DD increases aggregation and polydispersity. Increased NaCl enhances nanogel stability, reducing aggregation.	[194]
CS, TPP, NaCl	TPP	Particle size increases with higher-molecular-weight CS and higher CS concentrations: higher-molecular-weight CS (260 kDa) provides longer polymer chains, allowing for stronger cross-linking with TPP and the formation of larger particles; higher CS concentrations (0.1–0.2 wt%) supply more polymer molecules for cross-linking, further increasing particle size and stability. Ionic strength (NaCl concentration) slows down aggregation, following a power-law decrease with increasing monovalent salt levels. Higher TPP concentration accelerates aggregation, following a power-law increase. pH influences aggregation kinetics: Higher pH leads to stronger TPP binding and faster aggregation. Lower DD results in smaller, more uniform particles, while higher DD leads to rapid aggregation and greater polydispersity. Particles exhibit weak van der Waals interactions and aggregation is primarily governed by TPP bridging interactions. Monovalent salt (NaCl) addition enhances colloidal stability by reducing TPP bridging ability. Particle polydispersity can be minimized by lowering DD, adjusting ionic strength, and tuning TPP concentrations. Mixing methods significantly influence final particle size distributions, requiring precise control over TPP addition to avoid aggregation during mixing.	[195]
CS, TPP, NaCl, trehalose or mannitol or PEG	TPP	Formation of a bimodal size distribution: small particles (~40 nm) and larger aggregates (~250 nm) due to rearrangement after TPP addition. Storage for 4 weeks led to a slight increase in particle size, attributed to continuous rearrangement. The addition of NaCl influenced stability, with 150 mM NaCl preventing excessive aggregation while higher ionic strengths (500 mM) completely inhibited gelation. Nanoparticles showed positive zeta potential, confirming strong CS–TPP electrostatic interactions. Higher CS molecular weight led to larger nanoparticles and lower polydispersity was observed with very-low-molecular-weight CS. Freeze-drying and spray-drying methods were tested to improve stability, with trehalose proving to be the best cryoprotectant for preventing aggregation. Protein-loaded nanoparticles (BSA, OVA, HI) demonstrated good loading efficiency and size stability, with minor size increases post-loading. Chorioallantoic membrane assay confirmed CS–TPP nanoparticles were biocompatible, while free TPP induced hemolysis. Spray-dried nanoparticles aggregated, requiring cryoprotectants for stability. Zeta potential decreased with increasing TPP concentrations, indicating surface charge modification by TPP binding. Controlled addition of TPP produced smaller, more stable nanoparticles, while excess TPP led to aggregation and precipitation. Drug encapsulation tests demonstrated the feasibility of using CS–TPP nanoparticles for mucosal drug delivery applications.	[196]
CS, TPP, indomethacin	TPP	Forms indomethacin-loaded CS nanoparticles. Optimized using Box–Behnken experimental design. Optimal CS concentration: 0.6 mg/mL, TPP: 0.4 mg/mL, stirring time: 120 min. Particle size: 321–675 nm. Zeta potential: +25–+32 mV. EE: 56–79%. Drug release: 48–73%. Drug content: 98–99%. Release follows the Higuchi matrix model.	[197]
CS, TPP, ASA, PRO	TPP	ASA (hydrophilic) and PRO (hydrophobic) were loaded together for controlled drug release. EE and LC varied significantly based on formulation factors: - TPP concentration: Higher TPP led to lower ASA EE (from 80.1% at 1 mg/mL to 68.5% at 2.5 mg/mL) but increased PRO EE (37.2% to 62.0%). - pH influence: EE was highest in acidic (pH 3, EE: ASA 59.1%, PRO 43.4%) and basic (pH 8.6, EE: ASA 68.5%, PRO 62.0%) conditions and lowest at neutral pH 7. - CS molecular weight: Higher molecular weight improved ASA EE (from 60.1% at 210 kDa to 68.5% at 670 kDa) but slightly reduced PRO EE (from 69.9% to 62.0%). Release profiles: ASA released over 24 h, while PRO had sustained release up to 120 h. Particle size influenced by TPP concentration, ranging from 100–300 nm. Drug release rates depended on pH, with neutral TPP solutions leading to faster release. Potential application in restenosis treatment due to the anti-atherosclerotic and antioxidant properties of ASA and PRO.	[198]
CS, TPP, captopril, amlodipine, valsartan	TPP	EE: captopril (92 ± 1.6%), valsartan (91 ± 0.9%), amlodipine (87 ± 0.5%). Zeta potential: reduced from +52.6 ± 4.8 to +46.5 ± 5.2 mV after drug encapsulation. Particle size: below 100 nm, with homogeneous dispersion. Captopril-loaded NPs were smooth and regular, while valsartan-loaded NPs showed agglomeration due to temperature sensitivity. Captopril exhibited gradual release in a physiological buffer over 24 h, supporting extended drug delivery. Potential for improved oral bioavailability of antihypertensive drugs with reduced dosage and side effects.	[199]
CS, TPP, DOX	TPP	Efficient encapsulation of DOX, with 17% encapsulation efficiency. Encapsulation only occurs when DOX is complexed with TPP before nanoparticle formation. Encapsulation led to an increase in nanoparticle size from 143.2 nm to 397.0 nm. Nanoparticles persisted in cell culture for up to 6 days. Effective uptake into cancer cells within 30 min, persisting for 24 h.	[200]
CS, HA, butyrate, TPP	TPP	Sustained inhibition of reactive oxygen species release by activated neutrophils. Enhanced anti-inflammatory effects compared to free butyrate due to prolonged extracellular presence. Stable formulation: HA: CS weight ratio of 1:1 with CS fraction of acetylated units = 0.64. EE: ~70%. Sustained release profile: 45% released in the first 20 min, reaching 87% after 4 h. Mucoadhesive properties, making it a potential candidate for inflammatory bowel disease treatment via enema delivery. Resistant to cellular internalization, ensuring extended availability at the inflammation site.	[201]
CS, HA, TPP, VEGF or PDGF-BB, BSA or heparin sodium salt	TPP	EE: VEGF (94%), PDGF-BB (54%). BSA (20% *w*/*w*) and heparin sodium salt (0.1% *w*/*w*) enhanced growth factor stability and prolonged activity. Average particle size: ~200 nm with positive zeta potential (~+30 mV), reduced to +17 mV when VEGF and heparin sodium salt were included. In vitro release: VEGF was completely released within 24 h, while PDGF-BB showed sustained release over ~7 days. Stable in biological media (EMEM cell culture medium, water), but aggregated in PBS due to ionic strength. Non-toxic in cell culture models, with viability above 80% even at 1 mg/mL concentration.	[202]
CS, HA, TPP	TPP	HA coating enabled CD44-mediated uptake in macrophages, with clustering influencing internalization efficiency. Two preparative methods (A & B) affected nanoparticle properties: - Method A: Slow complexation, yielding more compact nanoparticles (~303 nm, −58 mV zeta potential). - Method B: Faster complexation, resulting in larger, more porous nanoparticles (~487 nm, −39 mV zeta potential). Nanoparticles shrunk in biological media (DMEM + 10% FBS), likely due to osmotic effects. Potential for targeted drug delivery to CD44-overexpressing cells.	[203]
CS, HA, TPP, DOX, miR-34a	TPP	EE: DOX (48.3%) and miR-34a (91%). pH-responsive drug release: Faster DOX and miR-34a release at acidic pH (~5.5, tumor microenvironment). Dual action mechanism: DOX induces tumor cell apoptosis, while miR-34a suppresses Bcl-2 expression and Notch-1 signaling, inhibiting cancer cell survival and migration. Enhanced in vitro cytotoxicity: HA-CNPs significantly reduced IC_50_ (~4.6× lower than free DOX). Superior in vivo efficacy: Significant tumor size reduction in a triple-negative breast cancer xenograft model with low systemic toxicity. Stable in biological media (RPMI 1640 + 10% FBS) for 3 days.	[205]
CS, TPP, HA, Alg	TPP	CNPs exhibit cationic surfaces, reducing circulation time and bioavailability. Coating with HA or Alg reduces protein adsorption and macrophage uptake, enhancing biocompatibility. HA-CNPs show targeted delivery potential due to CD44 receptor binding. Alg-coated CNPs exhibit larger sizes and stronger anionic charges. Protein corona composition varies: HA-CNPs adsorb fewer inflammatory proteins, making them less immunogenic.	[206]
CS, TPP, HA, NaNO_2_, HCl, NaOH	TPP	HA coating reduces cytotoxicity and stabilizes nanoparticles by preventing aggregation. HA-CNPs maintain cell viability and reduce oxidative stress by scavenging reactive species of oxygen. CNPs alone cause mitochondrial damage and induce LDH release, while HA-CNPs mitigate these effects. HA-CNPs significantly reduce macrophage activation and decrease inflammatory markers (TNF-α, IL-1β, NO). HA-CNPs show enhanced biocompatibility and lower immunogenicity compared to uncoated CNPs.	[207]
CS, SA, CaCO_3_, GDL, tilmicosin	Ca^2+^	Encapsulates tilmicosin, improving its bioavailability and sustained release over 48 h. pH-responsive drug release: controlled release with faster dissolution in acidic environments (beneficial for infected tissues). High EE (67.89%) and LC (23.33%), ensuring effective drug delivery. Enhanced antibacterial activity against *S. aureus* compared to free tilmicosin. Reduced cytotoxicity, biocompatible formulation without organic solvents or harsh chemicals. Effective against bovine mastitis: showed a 75% cure rate at normal dosage, outperforming commercial tilmicosin injection (50%).	[208]
CS, SA, TPP, SSD, acetic acid	TPP	Encapsulates SSD for controlled topical drug delivery. Initial burst release followed by sustained release, optimizing SSD bioavailability. Enhanced antibacterial activity against *S. aureus* and *P. aeruginosa*. Surface modification with SA improves nanogel stability and biocompatibility. In vivo efficacy: The optimized formulation accelerates burn wound healing compared to commercial SSD creams. Optimized formulation: 0.2% CS, 0.4% SA and 0.414% SSD.	[209]
CS, Alg, D-GDL, NaHCO_3_	Alg (poly-M or poly-G)	Gel formation occurs through controlled pH reduction, where CS becomes charged and interacts ionically with Alg. Gel strength increases as pH decreases from neutral to ~5. Poly-M Alg forms stronger gels with CS compared to poly-G Alg due to better charge matching. Higher ionic strength reduces gel strength due to electrostatic shielding effects. Homogeneous gels with tunable mechanical properties can be achieved by adjusting Alg composition and GDL concentration: - Higher GDL → stronger, stiffer gels with lower swelling and slower degradation. - Lower GDL → softer, more flexible gels with higher swelling and faster degradation Potential applications: Tissue engineering, controlled drug release, and biocompatible scaffolds.	[211]
CS, SA, TPP, CaCl_2_, mupirocin, acetic acid	TPP (for CS) CaCl_2_ (for Alg)	Two formulations: Alg-coated CS and CS-coated Alg nanogels. Encapsulates mupirocin, an antibiotic for controlled drug delivery. Enhanced antibacterial activity against *S. aureus* and *P. aeruginosa*. Controlled drug release: Mupirocin-loaded CS-coated Alg nanogels showed 96% release at 72 h, while Alg-coated CS nanogels released 70% at 72 h. In vitro and in vivo biocompatibility confirmed, with low cytotoxicity. CS-coated Alg nanogels exhibited superior antibacterial efficiency due to their positively charged surface enhancing cellular uptake.	[210]
CS, κ-carrageenan, TPP, acetic acid	TPP	Reduced particle size (150–300 nm) compared to CS–carrageenan nanoparticles without TPP (450–500 nm). Zeta potential decreased from +75–85 mV to +50–60 mV, improving nanoparticle stability. Production yield increased from 15–20% to 25–35%, and stability extended up to 9 months. Enhanced mucoadhesion, making it a promising system for mucosal drug delivery of macromolecules.	[212]
CS, κ-carrageenan, TPP, BSA, acetic acid	TPP	Stable nanoparticles (300 nm, +40 mV zeta potential) optimized for pulmonary and nasal drug delivery. BSA-loaded nanoparticles demonstrated controlled release with prolonged retention at epithelial surfaces. Stable in lysozyme-rich environments, showing potential for mucosal administration. Spray-dried microencapsulation with mannitol resulted in microparticles (2.3 μm, aerodynamic diameter 1.8 μm) for pulmonary protein delivery. Biocompatible formulation with no cytotoxicity and no inflammatory response in respiratory epithelial cells.	[213]
CS, sodium bicarbonate, 1-ethyl-3-(3-dimethylaminopropyl)carbodiimide)	-	Rapid gelation (<5 min) at 37 °C Strong bioadhesion due to catechol functionalization High mechanical stability (E = 90 kPa at 50% strain) Physiological pH and osmolality (~300 mOsm/mL). Potential for local drug delivery and cell encapsulation	[214]
CS, Alg, CaCl_2_, epidermal growth factor powder	CaCl_2_	The CMCS-Alg-4 hydrogel closely mimics the mechanical properties of native skin tissue. Porous 3D structure (~50–100 µm) supports cell attachment and proliferation. pH-responsive swelling and sustained EGF release for enhanced wound healing. High biocompatibility (>97% cell viability), low hemolysis (<1%), and strong antibacterial activity. Demonstrated accelerated wound healing in vivo, with faster re-epithelialization and granulation tissue formation.	[215]
CH-Su, Fe^3+^/Al^3+^/Ca^2+^, PBS	Fe^3+^/Al^3+^/Ca^2+^	Fe^3+^-cross-linked hydrogels have higher Young's modulus and longer degradation times than Al^3+^ and Ca^2+^-cross-linked hydrogels. Al^3+^-cross-linked hydrogels show the highest anti-adhesive effects in a rat model of peritoneal adhesion. Fe^3+^ hydrogels exhibit higher cytotoxicity due to Fe^3+^ release and pH reduction. Ca^2+^-cross-linked hydrogels degrade quickly (within ~1 h), while Fe^3+^ and Al^3+^ hydrogels degrade in ~46 h. Higher-molecular-weight CS increases hydrogel stability and decreases cytotoxicity. CH-Su/Al^3+^ hydrogels are most effective in preventing postoperative adhesions due to their balanced mechanical strength, degradation rate, and biocompatibility.	[216]
CS, TA, TPP	TPP	CS-based nanogels with high EE (91.41%) and uniform particle size (~394 nm). pH-responsive drug release: Faster release in acidic conditions (98% at pH 4.5) vs. controlled release at neutral pH (76% at pH 7.4). Enhanced antibacterial activity: TA-loaded CS nanogel increased antibacterial efficacy fourfold against *S. mutans*. Superior anti-biofilm properties: TA-loaded CS nanogel inhibited 72% of biofilm formation at 500 μg/mL, demonstrating effective biofilm penetration and disruption. Improved stability of TA: Nanogel encapsulation protected TA under light exposure and harsh environmental conditions.	[217]
CS, AAm, AAc, APS, halloysite nanotubes, HSiv, Fe(NO_3_)_3_·9H_2_O	Fe^3+^	Dual cross-linked hydrogel: ionically (Fe^3+^) and chemically (HSiv). Optimized formulation: CS-functionalized halloysite nanotubes at 2.5 wt%, AAc at 15 mol%, HSiv at 0.75 vol%, and Fe^3+^ ion soaking at 0.3 M for 16 h. High mechanical performance: tensile strength of 3.06 MPa, stretchability > 2000%, and toughness of 47.6 MJ/m^3^. Outstanding self-recovery: 97.9% at 200% strain, 91.5% at 1000% strain. Hydrogel retains ~80% water content, ensuring flexibility and resilience. Synergistic reinforcement: HSiv enables strong covalent bonding, while Fe^3+^ ionic coordination enhances elasticity and energy dissipation, leading to superior toughness and self-recovery. Potential applications: Load-bearing biomaterials, soft robotics, and next-generation flexible materials.	[218]

6PG-Na—6-phosphogluconate sodium salt; AAc—acrylic acid; AAm—acrylamide; Alg—alginic acid; APS—ammonium persulfate; ASA—aspirin; Bcl-2—B-cell lymphoma 2; BSA—bovine serum albumin; CH-Su—N-succinyl chitosan; CMCS—carboxymethyl chitosan; CNPs—chitosan nanoparticles; CS—chitosan; D-GDL—D-glucono-δ-lactone; DD—degree of deacetylation; DMEM—Dulbecco’s modified Eagle’s medium; DOX—doxorubicin; EE—encapsulation efficiency; EGF—epidermal growth factor; EMEM—Eagle’s minimum essential medium; FBS—fetal bovine serum; GDL—gluconolactone; HA—hyaluronic acid; HI—hemagglutinin inhibitor; HSiv—hyperbranched polysiloxane; IC_50_—half-maximal inhibitory concentration; IL-1β—interleukin-1 beta; ITC—isothermal titration calorimetry; LC—loading capacity; LDH—lactate dehydrogenase; MJ—megajoule; MPa—megapascal; miR-34a—microRNA-34a; NO—nitric oxide; NPs—nanoparticles; OVA—ovalbumin; PPi—pyrophosphate; PBS—phosphate-buffered saline; PDGF-BB—platelet-derived growth factor BB; PEG—polyethylene glycol; PRO—probucol; RPMI 1640—Roswell Park Memorial Institute 1640 (a cell culture medium); SSD—silver sulfadiazine; TA—tanshinone IIA; TEM—transmission electron microscopy; TPP –tripolyphosphate, TNF-α—tumor necrosis factor alpha; VEGF—vascular endothelial growth factor.

**Table 7:** Manufacturing CS gels through thermally-induced gelation.

Main Components	Cross-Linker	Characteristics	Reference
CS, G1-P, HCl, PBS	G1-P	Thermosensitive hydrogel forming in situ at ~37 °C. Non-reversible gelation. Gelation time depends on: - CS Mw: Low (~122 kDa) = faster (~3.5 min); High (~267 kDa) = slower (~15 min). - CS concentration: 1.5% *w*/*v* = ~2.5 min; 2% *w*/*v* = >20 min. - G1-P concentration: 0.40 mmol/g = ~2.5 min; 0.20 mmol/g = ~7 min. - pH effect: Higher G1-P → pH ↑ (~7.4) → faster gelation. Storage stability: At 2–8 °C, stable for at least 9 months, while CS–β -GP solutions gel in under a month. Suitable for injectable drug delivery with sustained release (days to weeks). Can be injected using thin needles (23–30 G) without excessive force.	[219]
CS, β-GP or G1-P or G6-P, HCl, Milli-Q water	β-GP, G1-P, or G6-P	Forms a thermosensitive hydrogel at ~37 °C. Gelation time depends on: - CS Molecular Weight (Mw): Low Mw (~122 kDa) = faster (~3.5 min); High Mw (~267 kDa) = slower (~15 min). - CS Concentration: Higher concentration (2% *w*/*v*) increases viscosity and prolongs gelation (>20 min), while lower concentrations (~1.5% *w*/*v*) lead to faster gelation (~2.5 min). - Gelling Agent Concentration: Higher β-GP/G1-P/G6-P (0.40 mmol/g) accelerates gelation (~2.5 min), while lower concentrations (~0.20 mmol/g) delay gelation (~7 min). - Polyol Size Effect: Larger polyol groups (G6-P > G1-P > β-GP) create stronger hydration layers, delaying gelation (β-GP ~2 min, G1-P ~12 min, G6-P ~24 min). - pH Influence: Higher gelling agent levels raise pH (~7.4), reducing CS ionization and promoting faster gelation. Storage stable for at least 9 months at 2–8 °C. Injectable (23–30 G needles) and enables sustained drug release (days to weeks). Biocompatible with mild inflammatory response reducing over time.	[220]
CS, β-GP, DBM, acetic acid	β-GP	Forms a thermosensitive hydrogel at ~37 °C. Hybrid scaffold with DBM improves mechanical strength (75% higher than pure hydrogel, 30% higher than DBM alone). Maintains high porosity and swelling capacity. Supports bone-derived mesenchymal stem cells with >90% viability. Enhances chondrogenic differentiation and GAG production. Potential for cartilage tissue engineering and osteochondral repair.	[221]
CS, β-GP, HA, β-TCP, silver or silver–palladium NPs	β-GP	Forms a thermosensitive hydrogel at ~37 °C. Enhanced bactericidal activity against gram-positive and gram-negative bacteria (*S. aureus* & *P. aeruginosa*) due to silver and silver–palladium incorporation. High biocompatibility with no cytotoxic effects recorded. Controlled degradation kinetics observed upon DMEM immersion. Potential application in biomedical and dental surgeries.	[222]
CS, thermosensitive polymer, NaHCO_3_	-	Injectable, thermosensitive hydrogel: Liquid at room temperature, gels within 50 s at 37 °C. pH-responsive gelation: NaHCO_3_ regulates the sol-gel transition without forming covalent bonds. Highly adhesive, biodegradable, and biocompatible. Promising for wound healing, tissue adhesives, and biomedical applications.	[223]

ß-GP—beta-glycerophosphate; ß-TCP—beta-tricalcium phosphate; CS-chitosan; DBM—demineralized bone matrix; DMEM—Dulbecco’s modified Eagle’s medium; G1-P—glucose-1-phosphate; G6-P—glucose-6-phosphate; GAG—glycosaminoglycan; HA—hydroxyapatite; Mw—molecular weight; NPs—nanoparticles; PBS—phosphate-buffered saline.

**Table 8:** Preparation of CS gels via electrostatic interactions.

Main Components	Characteristics	Reference
CS, dextran sulfate, acetic acid, NaCl	Physically cross-linked nanogel formed via electrostatic interactions between positively charged CS and negatively charged dextran sulfate. Reversible self-assembly mechanism: nanogel formation and disassembly controlled by salt concentration. Particle size: 350–580 nm, with a positive surface charge (30–58 mV). Stable for 40 days at 37 °C, indicating good colloidal stability. Potential applications in drug delivery, benefiting from controlled assembly and disassembly properties.	[224]
CS, SG, 5-FU	pH-sensitive hydrogel enabling controlled drug release 5-FU release: 60% at pH 7.4, 94% at pH 2.0). High mechanical strength (497.67 kPa at 84.65% strain, 9.14 kPa at 43.73% tensile strain). Synergistic antibacterial activity: 97.75% against *S. aureus*, 96.76% against *E. coli*. Biodegradable, biocompatible, and non-cytotoxic (97.57% cell viability). Potential applications in wound healing, tissue engineering, and drug release systems.	[225]
CS, two-component silicone elastomer, sulfate	Up to a fivefold increase in compressive modulus compared to pure CS scaffolds. Improved printing accuracy and scaffold stability. Tunable surface roughness and enhanced biodegradability. Supports cell adhesion and proliferation with no cytotoxicity. Minor impact of silk particle loading (up to 300% *w*/*w*) on ink rheology.	[226]
CS, Na_2_S_2_O_4_, monochloroacetic acid, Na_2_CO_3_, reactive dye (Supra rouge S-PX)	Effective against *E. coli*, *L. monocytogenes*, and *S. aureus*. Improved moisture management and breathability, with minimal reduction in air permeability (~10%). Maintained water vapor permeability while improving comfort properties. Altered fabric structure, increasing thickness and crease recovery angle. Potential application in antibacterial and functional textiles for medical use.	[227]
CS, carrageenan, laponite	Hydrogels exhibit high swelling ratios (5000%–6000%), with lower swelling at pH 5 compared to pH 2 and pH 7. Storage modulus of 5–10 kPa, increasing with CS content. Addition of laponite nanoclay improves mechanical stability and modulates swelling behavior. Hydrogels maintain their mechanical integrity across pH 2–7, making them suitable for drug delivery, tissue engineering, and bone regeneration.	[228]
CS, Alg, acetic acid	Dual-cross-linked hydrogel: Electrostatic + chemical bonding. pH-responsive: Suppresses drug release in gastric pH (2.2), and enhances release in intestinal pH (7.4). High drug loading efficiency (44%) for hydrophobic drugs (ketoprofen). Improved mechanical strength and stability compared to single electrostatic cross-linked hydrogels. Non-toxic (95% cell viability) and fully biodegradable within 4 days in enzyme solution.	[229]
CS, HA	Stable, non-sedimenting NPs were successfully formed. Medium molecular weight HA (257 kDa) resulted in stable NPs (~149 nm) with no sedimentation; lower molecular weight HA (176 kDa) also formed small NPs (~149 nm), but reduced viscosity and charge stability. CS glutamate NPs were more negatively charged than CS chloride NPs. Particle size varied, with the smallest being 149 ± 11 nm. Zeta potential depended on HA content and polymer mixing ratio. HA–CS NPs demonstrated non-toxicity at pH 5 and 7.4, suitable for oral formulations.	[230]
CS, HA, Zn^2+^	CS–HA polyelectrolyte complexes (PECs) stabilized using Zn^2+^ to improve colloidal stability in physiological conditions. Without Zn^2+^, PECs aggregated or dissolved in PBS, but stabilization enabled prolonged stability (≥35 days at room temperature). Smaller nanoparticles were achieved with Zn^2+^, suggesting cross-linking effects via coordination bonds. Zeta potential reduction with Zn^2+^ stabilization, indicating charge screening and improved electrostatic balance. Pre-stabilization (Zn^2+^ added during PEC formation) was more effective than post-stabilization.	[231]

5-FU—5-fluorouracil, Alg—alginate; CS—chitosan; HA—hyaluronic acid; NPs—nanoparticles; PBS—phosphate-buffered saline; PECs—polyelectrolyte complexes.

**Table 9:** Preparation of CS gels through hydrogen bonding interactions.

Main Components	Cross- Linker	Characteristics	Reference
CS, sodium Alg, citric acid	-	No covalent cross-linking; reversible and biocompatible. High swelling capacity in neutral and alkaline conditions. Potential for controlled drug delivery and bioactive molecule encapsulation. Environmentally friendly, non-toxic preparation method.	[234]
CS, orotic acid, 2,6-diaminopurine	-	Thermoreversible: Transitions between gel and sol states at room temperature and 80 °C. pH-responsive: Stable in acidic pH (2–3), dissolves in neutral/basic conditions. Self-healing ability, enabling structural recovery after damage. Potential for gastrointestinal drug delivery applications.	[235]
CS, micronized nystatin, succinic anhydride, diepoxy-functionalized siloxane, lactic acid, glycerine, nystatin	-	Hydrogel is obtained through hydrogen bonding and chemical cross-linking, ensuring a dual network structure. Hydrogels mimic soft tissue properties (elastic moduli <1 MPa), ensuring bioadhesion. Maximum swelling capacities: ~429% (pH 7.4) and ~471% (pH 4.2). Physical gels exhibit faster nystatin release (~57% in acidic pH, ~51% in pH 7.4), while chemically cross-linked gels have a more controlled release. Effective against *Candida* species due to CS bioadhesion and nystatin antifungal properties.	[236]
CS, TEPA, epichlorohydrin, Cr (VI)	TEPA	High adsorption capacity for Cr (VI) (~148.1 mg/g). Fast adsorption rate (equilibrium reached within 100 min). Mechanically strong, thermally stable 3D hydrogel. pH-sensitive adsorption, with maximum efficiency at pH 3.0. 100% removal efficiency at low Cr (VI) concentrations (0.02–1 mg/L). Highly reusable—maintains adsorption efficiency after five cycles.	[237]

CS—chitosan, TEPA—tetraethylenepentamine.

## Data Availability

No new data were created or analyzed in this study.
